# Spatiotemporal characterization of single-stranded DNA intermediates after UV irradiation: II. Rapid growth and effects of *recA* and *recJ*

**DOI:** 10.1371/journal.pgen.1012110

**Published:** 2026-05-14

**Authors:** Remy A. A. Ripandelli, Elizabeth A. Wood, Andrew Robinson, Antoine M. van Oijen, Michael M. Cox

**Affiliations:** 1 Molecular Horizons and School of Chemistry and Molecular Bioscience, University of Wollongong, Wollongong, Australia; 2 Department of Biochemistry, College of Agricultural and Life Sciences, University of Wisconsin-Madison, Madison, Wisconsin, United States of America; Michigan State University, UNITED STATES OF AMERICA

## Abstract

Irradiation of E. coli with UV light results in the formation of post-replication gaps and induction of the SOS response. Here, we investigate the dynamics of single strand gap formation and resolution in cells growing with a 50 min doubling time within specially designed microfluidic chips, making observations on fluorescent gap markers in individual cells over the course of 8 hours. In cells proficient for gap repair, irradiation with UV at 5 J/m^2^ triggers an immediate increase in the number and intensity of gap markers. Cells lacking *recF*, *recO*, or *recA* cannot repair gaps via the canonical gap repair pathway and exhibit elevated gap numbers and intensities over many hours. Major conclusions include: (1) Post-replication gaps are a major feature of DNA metabolism after UV irradiation. (2) The long-lived, high-intensity foci observed in *recF*, *recO*, or *recA* mutants are completely dependent on the RecJ nuclease. In the absence of other RecFOR pathway functions, RecJ-mediated enlargement of many gaps continues unabated for extended periods. (3) In the absence of RecA or other RecFOR pathway functions, cells accumulate repair intermediates that are bound by large numbers of SSB molecules. (4) In the absence of RecJ, other pathways, perhaps involving TLS, displace SSB in gaps. Our results also confirm, using a different experimental setup and protocol, that: (a) UV-related repair activities, including nucleotide excision repair of at least some lesions, may continue for multiple cell generations after exposure; and (b) we again see no evidence that RecF facilitates lesion skipping. Overall patterns of gap formation and resolution under rapid growth conditions are consistent with a burst of post-replication gap formation following UV irradiation, postulated in the accompanying report to rapidly trigger the SOS response.

## Author summary

When DNA is replicated in any cell, the replication fork often encounters obstructions in the form of DNA damage, tightly bound proteins, or template strand breaks. Large DNA lesions (large alterations in the structure of one or more adjacent nucleotides in the DNA) are often bypassed by the enzymes of the replication fork, leaving the damage behind in a single strand gap called a post-replication gap. This “lesion-skipping” process, postulated over 5 decades ago and subsequently studied by largely indirect methods, remains one of the least understood aspects of cellular DNA repair. In this and the accompanying report, we use some new methods to directly view the formation of post-replication gaps in bacterial cells after their DNA is damaged by ultraviolet radiation. A dedicated repair system addresses these post-replication gaps. Formation of such gaps is documented here as an important feature of the overall DNA repair process when cells are heavily damaged by ultraviolet radiation (UV). When enzymes that are part of that repair system are inactivated, the gaps accumulate. The patterns of gap appearance and accumulation in cells missing one or another of the repair enzymes allow us both to confirm previous ideas about the activities of these enzymes and also discover some new and unexpected features of these important repair systems.

## Introduction

In any organism, DNA replication forks routinely encounter barriers in the form of template lesions and bound proteins [1–8]. The consequences of such encounters varies with the type of barrier [1]. Encounters with large, bulky lesions on the template often trigger replisome lesion-skipping and the formation of post-replication gaps [1,9–11]. A bulky lesion in this sense refers to a nucleotide with a significant adduct or nucleotides that are linked together. The classic bulky lesions that trigger post-replication gap formation are the pyrimidine dimers generated by UV irradiation [12–33]. As the lesion is left in a region of single-stranded DNA, repair requires the installation of an undamaged complementary strand. This is predominantly accomplished via recombination, a path that is mechanistically complex but that avoids mutagenesis [1,10,34–36]. In bacteria, mutagenic translesion DNA synthesis [1,10,34–36] and a RecA-independent template switching pathway [1,37–39] provide alternative repair routes.

Recombinational repair of post-replication gaps in *Escherichia coli* is mediated primarily by the RecFOR recombination pathway (named for the first genes implicated in the pathway rather than a complex formed by the three) [1,4,25,40–44]. In this path, the RecR protein makes alternative and mutually exclusive complexes with the RecF and RecO proteins [45–51]. The RecFR complex functions to target the system to gaps requiring repair [1,52]. The RecOR complex loads RecA into the targeted gaps and can do so in vitro independently of RecF [45,46,48,52–55]. Although a stable and functional RecFOR complex has not been isolated, RecF and RecO must collaborate in this system. *In vitro* systems illustrating this collaboration have been established [48,56]. This might occur via a handoff of RecR from correctly positioned RecF to RecO, helping to activate the latter for RecA loading in a gap needing repair [1,57].

RecF must find its way to gaps requiring repair. An early suggestion that RecF bound specifically to gap ends [54,56] is not supported by detailed DNA binding studies that show RecF binding to all DNA structures with high affinity (*K*_d_ values all in low nM range) [49,50,58]. An alternative notion is that RecF interacts with proteins bound near the target gap. RecF does not interact with SSB [55,57,59]. However, RecF targeting might be affected by its recently revealed interactions with replisome components DnaN and DnaG [57]. Both RecO and RecF can be modified with fluorescent protein fusions at their C-termini without loss of function [9]. When this is done, the two proteins rarely co-localize. RecF is often found at the replication fork [9]. RecO forms foci much less frequently, usually at locations distal from the fork [9]. When RecF (but not RecO) is over-produced, it results in severe toxicity, reflected by replisome destabilization and loss [57]. This has resulted in the suggestion that RecF may not only target gaps requiring repair. It might be directly involved in their creation, perhaps via its interaction with DnaN [1,57]. This notion is directly tested in the present work and accompanying study [60].

The loaded RecA protein generates a joint molecule intermediate that connects the two daughter chromosomes behind the fork [1]. Resolution of this intermediate is crucial, since chromosome segregation at cell division becomes impossible if the daughter chromosomes remain linked. Under normal growth conditions, post-replication gap formation leading to this type of repair is frequent enough that cell death results if all resolution paths are blocked [17]. Triple deletion mutants that block all resolution paths, *rarA/ruvB/recG* or *rarA/ruvB/recQ*, exhibit synthetic lethality [17]. The synthetic lethality is suppressed by deletions of the *recF*, *recO*, or *recR* genes [17]. If RecA cannot be loaded into the gap and the joint molecule intermediates are not formed, the cell survives in the absence of joint molecule resolution pathways and finds other avenues for repair. Notably, deletion of another gene of the RecFOR pathway, the *recJ* gene, is as effective in suppression as is the deletion of *recF*, *recO* or *recR* [17]. This implicates RecJ in an early stage of the pathway leading to RecA loading and filament formation. RarA, the RuvABC proteins, RecG, and RecQ all provide avenues for joint molecule resolution late in the pathway [1,17].

The RecJ protein is a 5′ → 3′ single strand exonuclease. It readily and processively degrades ssDNA from 5′ ends [61–64]. At double strand breaks, it will degrade 5′ overhangs [61,62]. It will also act in concert with RecQ to process double strand ends, leaving long 3′ overhangs onto which RecA can be loaded [64,65]. In this regard, RecQ and RecJ can act as a backup for the RecBCD enzyme in double strand break repair [64–67]. However, RecQ and RecJ do not always act together in vivo [17,67]. As noted above, RecQ and RecJ appear *not* to act together in post-replication gap repair. RecJ carries out its early function in facilitating the loading of RecA protein in gaps even if RecQ is not present [17]. RecJ has been implicated in enlarging single-stranded post-replication gaps [10,36,68]. Recent work has begun to highlight the importance of the RecJ nuclease in gap repair. [10,17] However, the activity of RecJ in gapped DNA substrates has not been carefully explored *in vitro* or by direct observation *in vivo*.

In addition to the capacity of *recJ* deletions to suppress the lethality of mutant combinations that cannot resolve joint molecules behind the replication fork, several additional observations place RecJ at an early stage in post-replication gap repair. RecJ (along with RecF, O, and R) are not induced by the SOS response. However, SOS induction is generally delayed in cells lacking the function of any of the *recFOR* genes [69–77]. This is presumably because RecA filaments formed in post-replication gaps are the primary source of SOS induction. RecJ is also required for SOS induction in at least some genetic backgrounds [78].

The exploration of post-replication gap repair in vivo has been limited by an inability to visualize gaps in living cells. A new method to measure ssDNA globally in the *E. coli* genome [79] allows the measurement of differences in gap generation at different chromosomal locations and increases in ssDNA triggered by DNA damage. A new fluorescently labelled SSB which fully supports cell growth and replication even when wild type SSB is not present allows the visualization of individual gap generation [80].

When cells are subjected to UV irradiation in a laboratory setting, hundreds or even thousands of UV lesions are generated in each *E. coli* genome. DNA metabolism is immediately affected in many ways. Nucleotide excision repair (NER) begins to address the lesions immediately. NER is efficient but transcription-coupled repair ensures that some lesions are repaired more rapidly than others; some lesions linger for many tens of minutes [81–88]. Some lesions may linger for several hours [83,84,89]. As lesions are abundant, active replisomes will encounter intact lesions within seconds or a few minutes. The replisome may bypass them via lesion-skipping or halt. Although lesion-skipping is well-documented and should allow for continued replication, overall replication comes to a halt within about 5 min and remains stalled for a period of 20–80 min or more [90–93]. Recovery of replication elongation requires RecA [91,94,95], the RecFOR functions [23,90–92,96], RecJ [65], the nucleotide excision repair system [91,96,97], and PriA [98,99]. The molecular cause of the halt in replication is not entirely clear but presumably represents replisome encounters with damage. NER will produce transient breaks in the strand where a pyrimidine dimer is removed; a replisome encounter with that strand discontinuity should result in replisome collapse. Lesion-skipping occurs, but its contribution to the overall course of lesion amelioration relative to other processes has not been clear. To complement the accompanying manuscript [60], we present here another direct assessment of post-replication gaps *in vivo* after UV irradiation with multiple changes in parameters.

## Results

The goal of this study was to document the formation and processing of post-replication gaps in *E. coli* and begin to assess their contribution to DNA metabolism after UV irradiation. The current study complements the accompanying report [60] and was carried out for several reasons. The current study features a reduced cell cycle time (50 min vs ~ 3 hours in the accompanying report), allowing an assessment of the effects of growth rate. The current study also features an assessment of the effects of RecA and RecJ function. The effects of RecB are explored in the accompanying report. A comparison of two different experimental setups also allows for confirmation of major results and provides assurance that key observations are not dependent on observational approach.

To detect and visualize gaps, we used a new SSB-mTur2 fusion developed by Keck and coworkers [80] to image the ssDNA regions (i.e., replisome and gap). Previous SSB fusions studied to date [100–103] have the limitation that they place the fusion at the C-terminus, blocking the many interactions identified to date between SSB and other proteins of DNA repair and replication [104,105]. The loss of these interactions means that cells expressing only the fusion protein are inviable, and the cells must also express the wild type SSB to survive [100–103]. In the construct used here, the fluorescent protein domain is placed not at the C-terminus but within the intrinsically disordered linker (IDL) that connects the main OB fold domain with the small and conserved acidic C-terminus, between amino acid residues 148 and 149 [80]. *E. coli* cells grow normally when SSB-mTur2 is the only SSB expressed but exhibit modest sensitivities to some DNA damaging agents. Characterization of strains expressing only SSB-mTur2 in a variety of relevant genetic backgrounds can be found in the accompanying report.

With this tool, gaps formed during replication are detected during normal growth conditions. These are difficult to distinguish from less common lesion-containing post-replication gaps formed in response to replisome encounters with bulky lesions. In the accompanying study, we sought to minimize the replication background by using slow growth conditions. In this case, we instead recorded increases in SSB features after UV irradiation relative to this background. However, our conditions still limit detection of ongoing replication forks in WT cells. To increase the signal from post-replication gaps, we subjected cells to UV irradiation at 5 J/m^2^. This is a relatively low dose of UV, with modest effects on overall survival in most of the strains used in this study [106]. When cells are subjected to UV irradiation, lesions, mainly cyclobutane dimers and (6–4) photoproducts, are introduced into the chromosomal DNA at approximately 0.7 lesions per 100 kbp per J/m^2^ exposure [25,107,108]. At 5 J/m^2^, this would amount to about 160 lesions per *E. coli* genome, or one every 30,000 bp. For a replisome traveling at 1,000 bp s^–1^, most active replisomes should encounter a lesion within a minute after irradiation.

**Fig 1:**
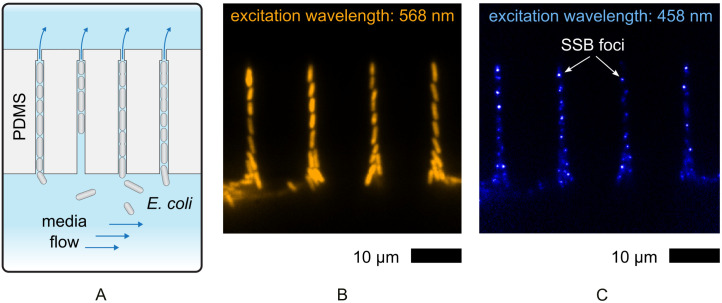
*E. coli* cells are trapped in a microfluidic chip and are monitored by fluorescence microscopy. A) A schematic depiction of the microchannels in the chip. The microchannels are only slightly larger than the cells, forcing the cells to grow in single file. A narrow section at the top of each microchannel prevents the cells from exiting but allows for continuous flow of nutrients. B) A microscopy image showing cells trapped in the microchannels. The cells constitutively express mKate2 in the cytosol, enabling automatic detection, segmentation and tracking of cells. C) The same field of view showing SSB-mTur2 signals. Foci form where SSB-mTur2 binds to ssDNA gaps. The number of foci is indicative of the number of (optically resolvable) SSB binding sites on the chromsome. The intensity (brightness) of each focus is indicative of the number of SSB proteins bound to DNA at each site. Cells are wild type with the exception of the incorporation of the gene encoding SSB-mTur2.

In the present study, timelapse microscopy data were collected from 10 *E. coli* MG1655 strains, including wild-type and 9 knock-out mutants with combinations of *ΔrecF*, *ΔrecO,* Δ*recJ,* and *ΔrecA*. Single cells of each strain were loaded in single file into microchannels within a custom microfluidic chip (Figs 1 and S1–S3), based on the mother-machine concept [109–111]. Fluorescence images were recorded in timelapse to monitor signals from fluorescently tagged single-stranded-binding (SSB) protein, as well as from mKate2 expressed in the cytosol, which enables automated detection and tracking of cells throughout the measurements. The chip design provides a continuous supply of growth medium through the microchannels, ensuring consistent delivery of nutrients and oxygen to the cells. To reduce the number of active replisomes present in each cell, nutrient-poor M9 medium with added glucose was used. However, the chip was maintained at 37 °C, resulting in more rapid growth than that observed in the accompanying study. Quantitative single-cell data was acquired before and after the UV treatment. For each time-lapse recording, approximately 500–1000 cells were monitored, with data collected for 100 ms, every 5 min for 4 hours prior to UV treatment and every 3 minutes for four hours post treatment. The single-cell data included the number of SSB sites and SSB proteins per cell and per frame, acquired before and after a UV exposure of 5 J/m^2^ (Fig 1C). For each individual cell, the results from the UV-treatment were normalized to results acquired from the same cell prior to the UV exposure. A total of 32 experiments were conducted (generally 3–4 per strain), resulting in about 4,000,000 segmented cells with their corresponding SSB characteristics. The image data were processed and analyzed using our in-house artificial intelligence pipeline [109].

The data collected during this microfluidic study are presented in two forms. The UV-irradiation data (presented in the main text) show the relative numbers and intensities of SSB foci before and after the UV treatment. The absolute intensity values for each individual repeat experiment are presented in the supplementary information (S4 to S13 Figs). Cell cycle data (presented in the supplementary information; S1 Text) show the numbers and intensities of SSB foci in untreated cells throughout normalized cell cycles. Cell cycle periods were similar for all strains with a median of about 50 minutes per cycle (S14 to S16 Figs). The SSB focus intensity values in the cell cycle diagrams show that *rec* mutant strains tended to have slightly elevated focus intensities when compared to the *rec*^*+*^ strain (S20 to S22 Figs). For this reason, we normalized the focus intensities in the post-UV data to the pre-UV values for each strain.

**Fig 2:**
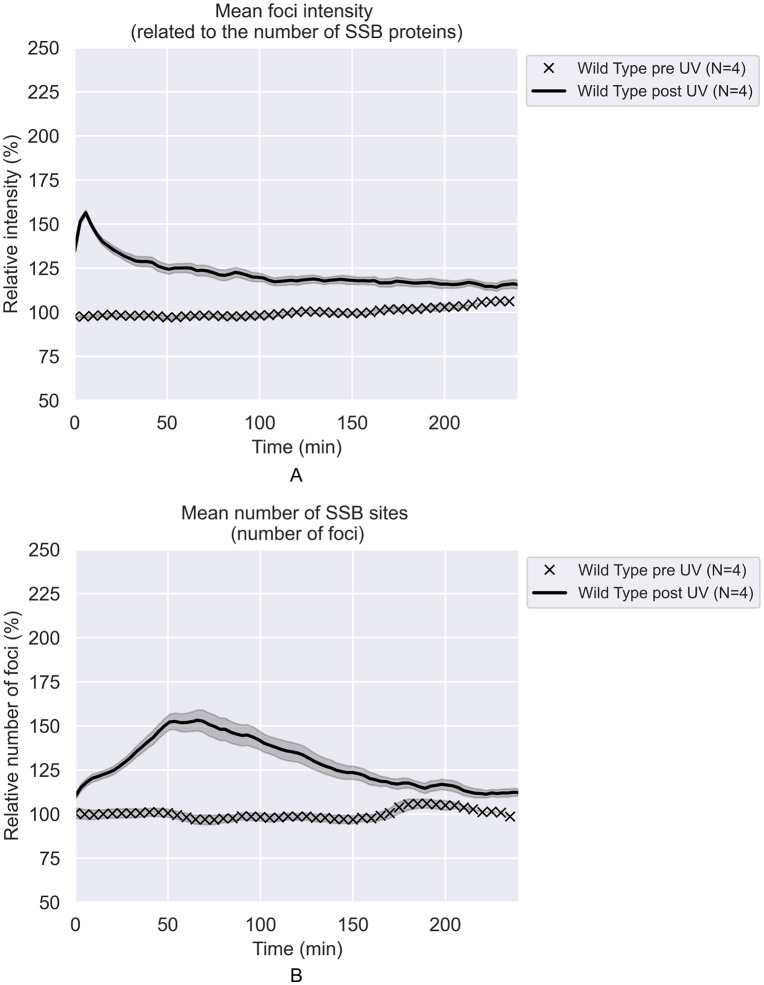
The effects of 5 J/m^2^ UV treatment on SSB foci in repair-proficient *E. coli.* All values are expressed relative to the average of pre-UV values. Each curve is the weighted average over the repeats **(N)**. The shaded areas represent the standard deviations. **A)** Relative intensities of SSB foci vs time after irradiation. **B)** Relative number of SSB foci per cell vs time after irradiation. Cells are wild type with the exception of the incorporation of the gene encoding SSB-mTur2.

**Fig 3:**
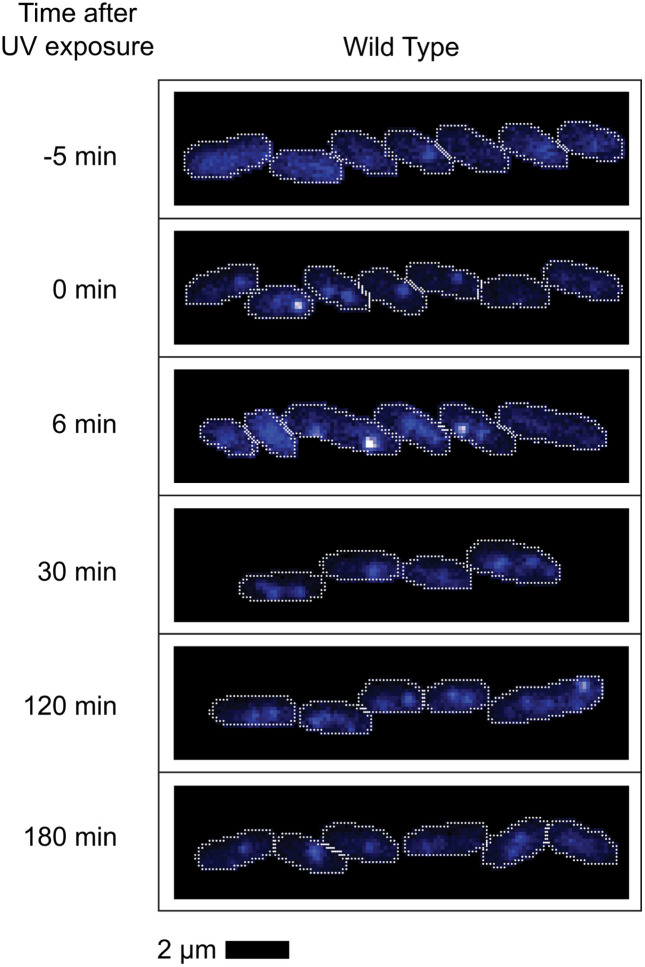
Example fluorescence images of SSB-mTur2 signals in repair-proficient cells taken at key moments following UV irradiation. White lines indicate cell outlines assigned by AI-driven cell segmentation. Cells are wild type with the exception of the incorporation of the gene encoding SSB-mTur2.

The results with repair-proficient cells (*rec*^+^; expressing SSB-mTur2 and mKate2, but otherwise wild type) are presented in Fig 2. Following UV irradiation, the SSB foci grow more intense and additional foci form, indicating an increase in the total number of SSB-bound ssDNA gaps. The number of foci increases for just over one hour and then begins to decline, possibly due in part to transient filamentation. This increase is greater and more rapid than observed for the WT cells growing more slowly in the accompanying report, as is the subsequent decline. This may reflect a more robust repair environment at the higher temperature used here. Focus numbers approach those of untreated cells three hours after treatment. The average intensity of foci increases rapidly after UV exposure, but peaks at about 6 min and then declines to a lower level, albeit somewhat greater than seen in the untreated cells. This level is maintained for at least 4 hours (Figs 2 and 3).

These patterns are consistent with the following scenario. Upon the introduction of UV damage, active replisomes quickly encounter template pyrimidine dimers that trigger post-replication gap formation. As some of these lesions will be repaired via nucleotide excision repair, replisomes may also encounter transient template discontinuities at sites of ongoing NER. The resulting double strand breaks and processing by RecBCD may also account for some of the SSB foci observed. Replisomes that undergo lesion skipping may continue and encounter additional lesions that trigger additional gap formation for an extended period of time. Since normal rates of replication normally resume in wild type cells within 20–80 minutes after UV irradiation [65,90,96,112,113], the continued higher levels of gap generation suggest an ongoing process, addressed below in the Discussion. The transient peak in focus intensity values would be expected if many replisomes encountered lesions initially more-or-less simultaneously (or a single replisome encountered multiple lesions in rapid succession), gaps were formed and enlarged, followed by elimination of the signal as RecA was loaded into the gaps and displacing the labelled SSB. Subsequent lesion encounters would be less synchronized and lead to a kind of slowly declining steady state in the average brightness parameter. We show later that this initial peak in focus intensity is not due to RecJ action.

**Fig 4:**
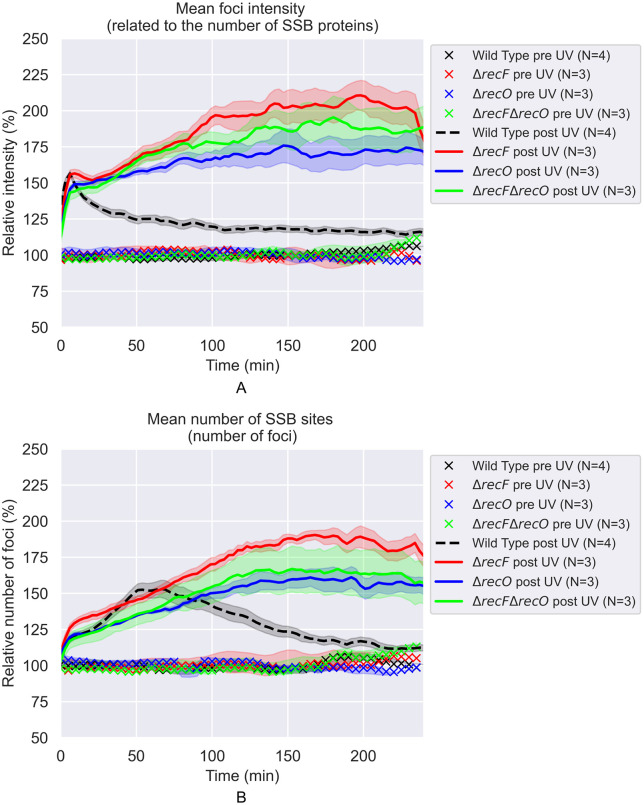
The effects of 5 J/m^2^ UV treatment on SSB foci in Δ*recF*, Δ*recO*, and Δ*recF* Δ*recO* cells. All values are expressed relative to the average of pre-UV values. Each curve is the weighted average over the repeats **(N)**. The shaded areas represent the standard deviations. **A)** Relative intensities of SSB foci vs time after irradiation. **B)** Relative number of SSB foci per cell vs time after irradiation.

**Fig 5:**
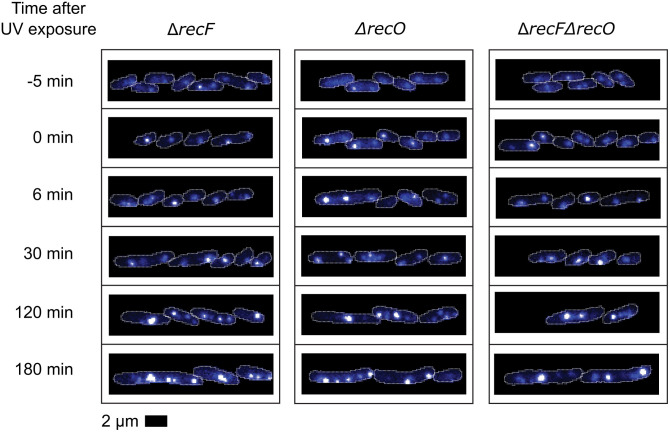
Example fluorescence images of SSB-mTur2 signals in *ΔrecF*, *ΔrecO* and *ΔrecF-ΔrecO* cells taken at key moments following UV irradiation. White lines indicate cell outlines assigned by AI-driven cell segmentation.

**Fig 6:**
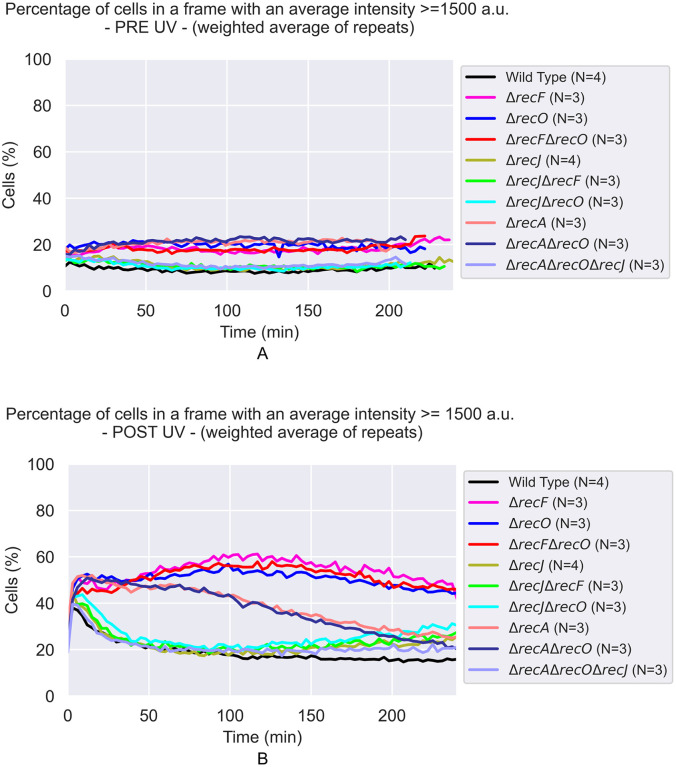
Percentage of cells containing at least one SSB-mTur2 focus with an intensity above 1,500 a.u. This threshold corresponds to approximately 150% of the average pre-UV treatment value. Each curve is the weighted average over the repeats. **A)** Pre-UV treatment of each strain. **B)** Post-UV treatment of each strain.

That many of the new SSB foci reflect lesion skipping to generate post-replication gaps can be seen in the effects of the RecF and RecO proteins. Elimination of the function of RecF, RecO, or both RecF and RecO has a dramatic effect on these patterns. Under the conditions of this study the effects are essentially identical for all three strains. In each case, the average focus intensities in these repair-deficient strains continue to increase for at least 4 hours after UV treatment (Figs 4A and 5), in contrast with the rapid reduction observed for the repair-proficient (*rec*^*+*^) strain. For each of the repair-deficient strains, the number of SSB foci in each cell increases throughout the measurements, possibly due in part to increased filamentation in the mutant strains (Fig 4), in contrast with the repair-proficient strain, which exhibited a decrease in SSB foci in the period beginning 60 min after treatment. The absence of the RecFOR proteins evidently prevents loading RecA into gaps created by replisome lesion-skipping. The result is that gaps are formed and enlarged, and this continues as no RecA is loaded into the gaps to displace the SSB. The increase in average focus intensity reflects the gradual formation of very bright foci in a subset (about 50–60%) of the cells (Fig 6). In contrast, the number of SSB foci increases for almost all of the cells being monitored (Figs 4B and 5).

Notably, the results for cells lacking RecF are the same as for cells lacking RecO. There is no evidence in this work that RecF is involved in the lesion-skipping process that creates the gaps, since gaps are being created at comparable rates in the absence of RecF function. However, the absence of RecF is still an impediment to the repair of many of the gaps that are formed. We note that the differences seen in cells lacking *recF* vs *recO* in the accompanying study, carried out at a lower temperature, was not evident here.

**Fig 7:**
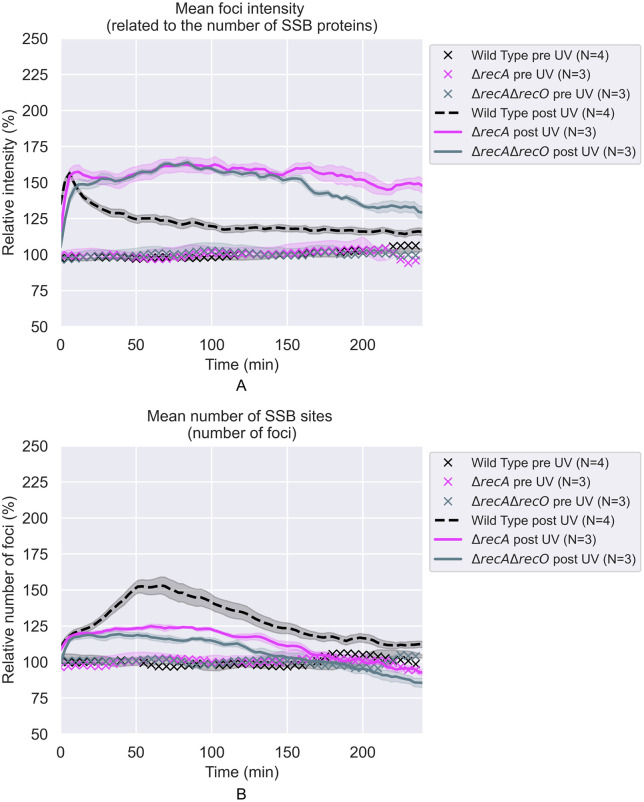
The effects of 5 J/m^2^ UV treatment on SSB foci in Δ*recA* and Δ*recA* Δ*recO* cells. All values are expressed relative to the average of pre-UV values. Each curve is the weighted average over the repeats **(N)**. The shaded areas represent the standard deviations. **A)** Relative brightness (intensities) of SSB foci vs time after irradiation. **B)** Relative number of SSB foci per cell vs time after irradiation.

**Fig 8:**
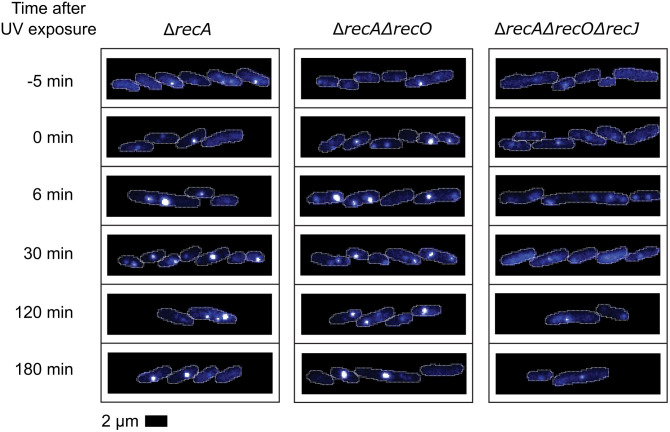
Example fluorescence images of SSB-mTur2 signals in *ΔrecA*, *ΔrecA ΔrecO* and *ΔrecA ΔrecO ΔrecJ* cells taken at key moments following UV irradiation. White dots indicate cell outlines assigned by AI-driven cell segmentation.

When RecA function is eliminated, the results are similar to those seen in the absence of the RecF or RecO function. However, the response is somewhat muted compared to that in the *recF* and *recO* mutants. Both the number of foci and their and average intensities increase. However, the increases are not as large for the *recA* mutant as for the *recF* and *recO* mutants (Figs 7 and 8). Very intense foci are again formed in a subset (~40–50%) of cells (Figs 6, S4, S11, and S12). The muted response may simply be due to the greater sensitivity of cells lacking RecA to DNA damage than is the case for *recF* or *recO* mutants. Cells lacking RecA largely do not recover replication after UV irradiation [114] and suffer extensive genomic degradation [91,94,95]. Supplementary tiff files to be found online (at dx.doi.org/10.6084/m9.figshare.30814493) show that cells lacking RecA typically filament and/or stop growing. Replisomes that encounter intact lesions will create post-replication gaps but they will not be resolved readily by recombination. Replisomes encountering template discontinuities will collapse but the double strand breaks will not be repaired. Many of these events will offer DNA ends for exonuclease digestion, processes that may eliminate the substrates for SSB binding. Cells lacking RecA are severely UV sensitive, as measured by many labs, with one example here [115].

**Fig 9:**
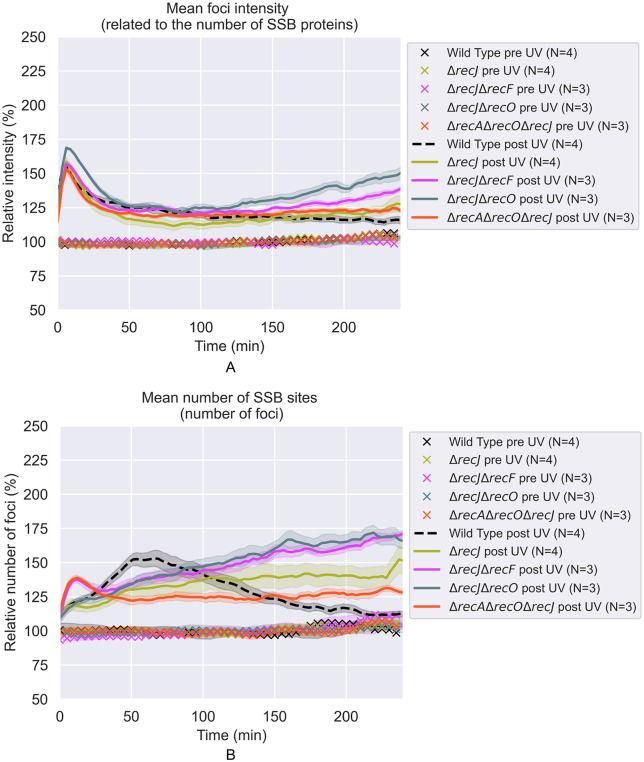
The effects of 5 J/m^2^ UV treatment on SSB foci in *ΔrecJ*, *ΔrecJ-ΔrecF*, *ΔrecJ-ΔrecO*, and *ΔrecJ-ΔrecA-ΔrecO* cells. All values are expressed relative to the average of pre-UV values. Each curve is the weighted average over the repeats **(N)**. The shaded areas represent the standard deviations. **A)** Relative brightness (intensities) of SSB foci vs time after irradiation. **B)** Relative number of SSB foci per cell vs time after irradiation.

**Fig 10:**
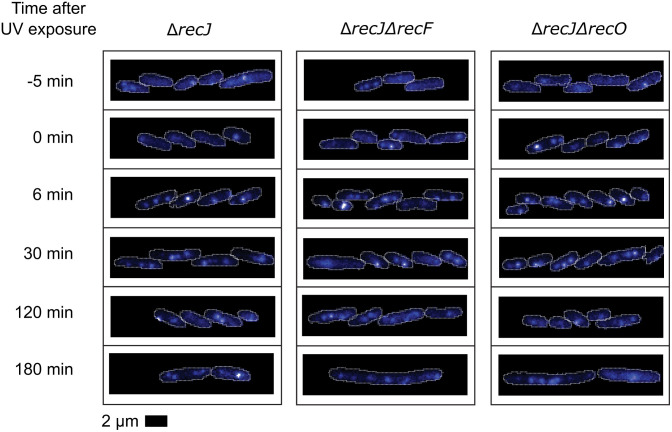
Example fluorescence images of SSB-mTur2 signals in *ΔrecJ*, *ΔrecJ ΔrecF* and *ΔrecJ ΔrecO* cells taken at key moments following UV irradiation. White lines indicate cell outlines assigned by AI-driven cell segmentation.

**Fig 11:**
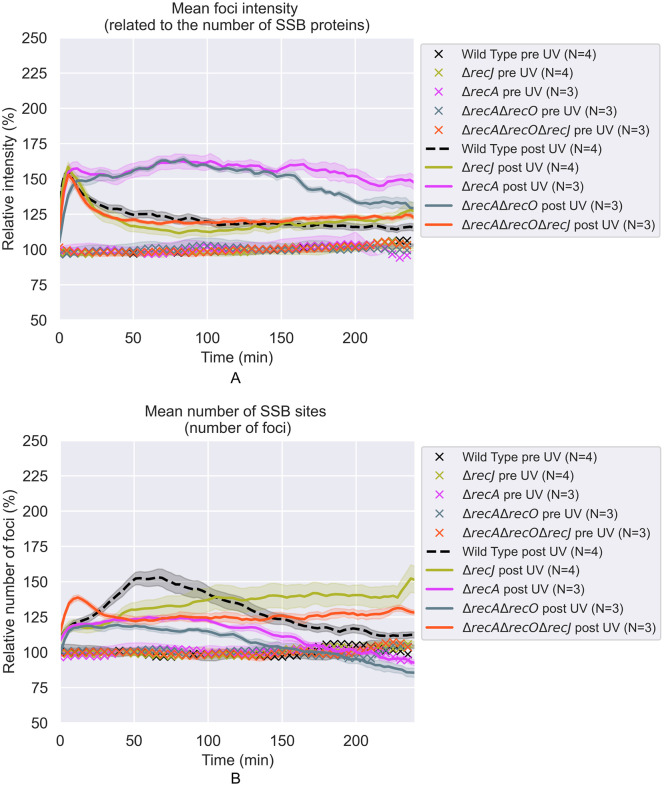
The effects of 5 J/m^2^ UV treatment on SSB foci in Δ*recJ*, Δ*recA*, Δr*ecA* Δr*ecO*, and Δ*recA* Δr*ecJ* Δ*recO* cells. All values are expressed relative to the average of pre-UV values. Each curve is the weighted average over the repeats **(N)**. The shaded areas represent the standard deviations. **A)** Relative brightness (intensities) of SSB foci vs time after irradiation. **B)** Relative number of SSB foci per cell vs time after irradiation.

**Fig 12:**
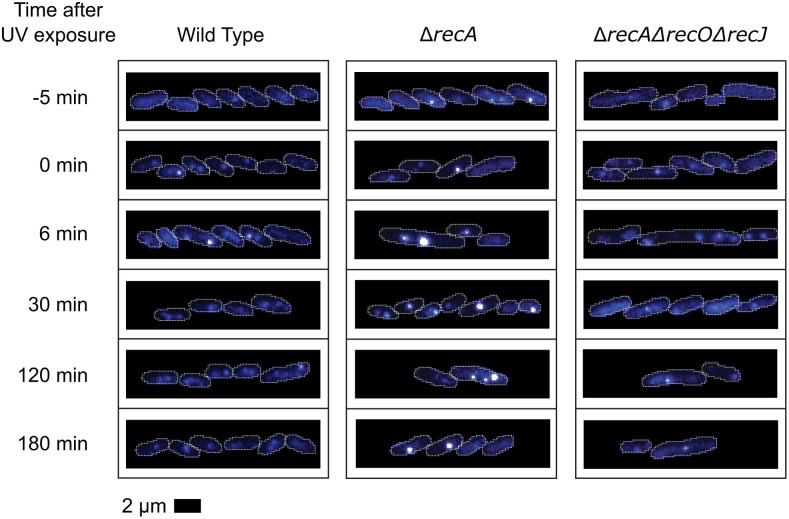
Example fluorescence images of SSB-mTur2 signals in repair-proficient, *ΔrecA, and ΔrecA ΔrecO ΔrecJ* cells taken at key moments following UV irradiation. White lines indicate cell outlines assigned by AI-driven cell segmentation.

Courcelle and Hanawalt [65] and Lloyd and colleagues [116] previously observed that RecJ-dependent DNA degradation increased when one of the RecFOR proteins was missing. The first of these studies was part of the evidence supporting a proposal for RecFORA function in protecting stalled replication forks [117]. The latter study linked the RecJ-mediated degradation with other processes surrounding replication restart. Here, we examined RecJ function in the context of post-replication gaps. The effects of losing the function of the RecJ exonuclease can be seen in Fig 6, and more results are shown in Figs 8–12. When the *recJ* gene is deleted, the large increase in average focus intensity is eliminated. With respect to focus intensities, the *recJ* deletion strains, with or without additional deletions in *recF*, *recO*, or *recA*, behave like repair-proficient cells; there is a spike in average intensity peaking at 6 min followed by a rapid decline. This indicates that this initial spike is not dependent on RecJ. The increases in gap numbers seen in the *recF*, *recO*, and *recA* deletion strains is still observed with the *recJ* deletions. However, the large increase in the intensity of many foci seen over multiple hours, which would be consistent with increases in gap size, is almost completely dependent on RecJ (Figs 10-12).

## Discussion

Many approaches have been used to gain an understanding of what happens when replisomes skip over lesions and generate post-replication gaps [1]. We present here a direct visualization of gap formation and resolution, using a labeled SSB that ably supports cell growth on its own and should be present in every gap large enough to include an SSB binding site. The results allow multiple conclusions. (1) In wild type cells and most mutants, a short but transient increase and then decrease in average focus brightness occurs immediately after UV irradiation. The increase in this early peak is not RecJ dependent. Under these conditions, where growth is more rapid than in the accompanying report, we speculate that some replisome coordination is present initially that leads to the formation of multiple nearby gaps. The short burst in brightness occurs in the first 5–10 min, a period that corresponds to the short time after UV irradiation in which replication continues prior to its extended halt [82]. The short burst in brightness also corresponds to a rapid early increase in foci number, before the increase levels off to a slower rate. This coordination may simply reflect the fast encounters of many active replisomes with intact UV lesions immediately after irradiation. This coordination would dissipate at later times as new *oriC*-dependent replication initiation events occur. (2) In cells lacking RecF or RecO, unresolved post-replication gaps lead to extensive formation of SSB foci. Very bright foci result and increase in number over a period of hours. These bright foci are almost completely RecJ dependent. (Figs 4,7–8, and 11). This result links at least some and perhaps the majority of the RecJ-mediated DNA degradation detected previously in *recFOR* mutants [65,82] to expansion of post-replication gaps. (3) Gaps are still readily formed without RecF present, indicating that RecF has little or no role in facilitating lesion-skipping. The replisome destabilization effects of RecF over-production [57] thus reflect an effect that does not apply to normal levels of RecF. (4) Finally, although replication resumes at normal rates in wild type cells within 20–80 min after UV exposure [65,90,96,112,113] (and perhaps faster in cells irradiated at our low dose of 5 J/m^2^), an increased number of ssDNA gaps is observed for several hours, even in repair-proficient cells. This likely reflects the presence of lingering UV lesions that have not been addressed by NER [83,84,89].

Conclusion 1 is consistent with the burst in post-replication gap formation postulated to trigger SOS induction rapidly after UV irradiation in the accompanying report [60]. Conclusion 2 highlights the role of RecJ in gap expansion. Conclusions 3 and 4 reinforce additional observations made in the accompanying paper [60] and make it unlikely that observed effects are anomalies related to experimental conditions or observation methods employed. Gaps are clearly formed after UV irradiation in the absence of RecF, indicating that prior suggestions that RecF might facilitate replisome lesion skipping [57] are not borne out (or at a minimum, RecF is not required). In addition, at least some UV lesions persist in these cells for many hours after the lesions are introduced, with post-replication gaps continually formed as replisomes encounter new ones. This indicates that the nucleotide excision repair pathway (NER) has some limitations when confronted by the sudden introduction of hundreds of lesions.

Transcription-coupled repair targets the NER system first to lesions in the transcribed strand of actively transcribed genes [85,86]. Lesions in other locales are repaired more slowly [85–88]. Our results suggest that a significant number of UV lesions, perhaps in regions that are not undergoing transcription at all, remain unaddressed by NER for a considerable period of time, consistent with previous observations made with different methods [83,84,89]. This highlights the importance of replisome lesion-skipping and resolution of the resulting post-replication gaps. It is important to reiterate that RecFORJ-mediated gap repair does not actually repair the lesion. After the gap is eliminated, the lesion must eventually be resolved by NER. As cells with wild type backgrounds are dividing about every 50 min under these conditions (S14 Fig) the persistence of the elevated level of foci over multiple cell generations (or in some cases multinucleate filaments) suggests the following: once recombinational DNA repair has been completed, removal of at least some lesions by nucleotide excision repair may not occur prior to the next replication cycle. The lesion itself might sometimes, perhaps often, survive to be encountered by a new replisome so as to trigger the formation of post-replication gaps in multiple cell cycles. Some of the 458 nm tiff movies, available online (see data availability) show, dividing cells with new foci appearing.

When an *E. coli* cell is subjected to UV irradiation, studies to date indicate a complex response occurs. Pyrimidine dimers, bulky lesions that are the classic arbiters of lesion-skipping and post-replication gap generation, are introduced at high levels in the chromosome. Repair of some of these lesions by nucleotide excision repair is likely to be initiated immediately. At UV doses of 25–30 J/m^2^, replication halts in wild type cells for about 20–80 min before resuming at normal rates [65,90,96,112,113]. Even at 5 J/m^2^, most replisomes are likely to encounter intact lesions in the first half minute. The initial burst in foci brightness in wild type cells and those lacking RecJ could reflect the initial encounters of replisomes with intact lesions that trigger lesion skipping. This burst is not observed under the slow growth conditions in the accompanying report, perhaps because fewer replisomes are active at the time of UV irradiation. The same initial burst coupled with a large and longer-term increase in SSB foci seen in cells lacking RecF or RecO indicate that many and perhaps most of the new foci represent post-replication gaps. The halt in DNA replication could be precipitated by replisome collapse after encounters with strand discontinuities created by ongoing NER or encounters with barriers that cannot be bypassed. However, multiple post-replication gaps appear to be created in the few minutes prior to the replication halt. Those gaps could then trigger SOS, increasing the availability of NER functions. RecF and RecO (and RecJ) are important for SOS induction, indicating that RecA filaments loaded into post-replication gaps represent a key modulator for the SOS response. The SOS response will thus be induced in at least some but probably not all cells [118–122]. Post-replication gap repair via RecA-mediated recombination will be triggered in most of these gaps. In wild type cells, this process predominates over the translesion DNA synthesis alternative for gap closing, although TLS can become more important if recombination is not available [10,34].

As already noted, RecF, RecO, and RecJ are all needed to efficiently resume replication and induce SOS. Deletions in the genes encoding any one of these proteins will suppress the synthetic lethality of the *rarA ruvB recG/Q* triple deletion strains [17], indicating that they all have a role early in the process of post-replication gap repair. All of this suggests a key role for RecJ, in addition to RecF, RecO, and RecR, is the loading of RecA protein into a post-replication gap. RecQ is not essential for this process since RecJ can facilitate RecA loading even when RecQ is not present [17]. Thus, gap enlargement by RecJ, perhaps in concert with a helicase other than RecQ, appears to be a critical process in the early stages of post-replication gap repair leading to RecA loading and filament formation. There is some gap resolution in the absence of any of the RecFORJ proteins. TLS may act to eventually close those gaps that are not successfully resolved by recombination. When RecF or RecO is absent, RecJ enlarges gaps and RecJ or other proteins may somehow facilitate RecA loading in some gaps or may facilitate a RecA-independent path [123].

Replisome lesion-skipping to generate lesion-containing post-replication gaps occurs mainly when replisomes encounter large bulky lesions such as nucleotides with substantial adducts or nucleotides that are linked to each other [1]. Classically, the lesions used to explore post-replication gap repair are the pyrimidine dimers inflicted by UV irradiation [12–33]. Recombinational DNA repair does not repair the lesion itself. Instead, it provides an undamaged complementary DNA strand to pair with the lesion-containing strand. Actual repair via nucleotide excision repair can then use the undamaged complement to facilitate accurate repair. Although ongoing replication forks are halted shortly after UV irradiation, origin-dependent replication initiation continues [82,95]. The results presented here suggest that the time requirements for recombinational repair following replication and lesion-skipping may not always provide an adequate time window for subsequent NER prior to arrival of the next replication fork.

The work presented here offers some new insights and provides a baseline for future exploration to answer a wide range of additional questions. A few obvious ones are considered here. If the actual UV lesions are eventually eliminated by NER, then the effects of a *uvrA* mutation to eliminate NER in these cells will be of interest. We might anticipate that elevated levels of gap formation after UV might extend for even longer periods in such cells. The extent to which double strand breaks contribute to focus formation after UV may be revealed using mutants that eliminate one or more of the pathways by which double strand ends can be processed (recBCD or recQJ), with one initial set of observations offered in the accompanying study [60]. Activities that might partner with RecJ to promote gap enlargement also require further consideration and exploration. The helicase partner often assigned to RecJ, the RecQ helicase [64,65,124], does not function with RecJ in post-replication gap enlargement, or at least RecJ functions early in gap processing even when RecQ is absent [17,67]. In recombinational repair of post-replication gaps, RecJ acts early; RecQ and RecG act late after joint molecules are formed [17]. However, work to date *in vitro* suggests that RecJ cannot efficiently enlarge gaps on its own [62–65]. Activation by SSB or partnership with some other helicase still to be discovered may be necessary.

## Methods

The fluorescence microscopy data reported in this study were acquired in timelapse mode and made use of a custom microfluidic chip that enabled cells to be cultured in single file within microchannels [109]. For each measurement, the chip was loaded with cells from a single genetic background. In each measurement, cells were monitored for a period of 4 h under standard growth conditions, before irradiating with 5 J/m^2^ UV light. Cells were monitored for a further 4 h after treatment.

### The microfluidic chip

For this study we used a single-cell fluorescence microscopy approach in which we monitored live bacteria in an in-house made microfluidic chip. The chip design was based on the so-called mother machine that was invented in 2010 and subsequently optimized (Figs 1 and S1) [110,111]. With this chip, *E. coli* cells can be loaded and trapped in microchannels with a cross section roughly that of a bacterium grown in minimal media. The dimensions of the channels are approximately 1 µm in width and 0.8 µm in height and 15 µm in length (see S2 Fig). Consequently, the cells are lined up along their long axis, with the cell that loads first being referred to as the mother cell. During the experiment, this trapped mother cell divides, pushing one of the two daughter cells out of the channel. The chip design allows active flow through the microchannels for easy cell loading and a constant supply of nutrients and oxygen [109]. The microfluidic chip contains hundreds of microchannels that can be monitored in timelapse using a microscope with a programmable microscope stage.

### The chip preparation

To produce the chip, a master mold was designed and fabricated in house. The circuit was patterned on SU8 negative photoresist (SU8–2002, SU8–2075, Kayaku Advanced Materials) using a mask-less UV writer (Durham Magneto Optics Ltd MicroWriter ML3 Pro 365 nm; see centre of S2 Fig). The fabrication was done in a class 100 (ISO 5) clean room. The microfluidic chip was built by casting polydimethylsiloxane (PDMS) (Sylgard). The PDMS was prepared as per the manufacturer’s instructions and poured onto the master mold. Subsequently the PDMS was cured at 150 °C and stripped from the master. It was then trimmed to fit a coverslip and punctured to create the inlet and outlet holes. Finally, the PDMS chip was washed and sonicated in detergent (TRITON X-100) and isopropanol alcohol (IPA) to reduce the toxicity of the PDMS for bacteria and to sterilize the chip [125]. After drying at 80 °C overnight, the chip was attached to a glass slide using a plasma treatment. The inlet and outlet tubes are connected, and the chip was loaded with a minimum media solution supplemented with 1–2% Bovine Serum Albumin (BSA). The BSA solution was passed through the chip for approximately 30 minutes to prevent the bacterial cells from sticking to the glass surface during the experiment. The details of the master mold fabrication and the chip preparation are described in a recently published methods paper [109].

### Strains and culture preparation

A set of 10 *E.coli* strains were used, all derived from MG1655. All contained an allele expressing an SSB-mTur2 fusion (to visualise SSB-loaded ssDNA gaps) and a plasmid expressing mKate2 (as a cytosol marker to for detection and segmentation) [80,126]. The 10 strains included a repair-proficient (*recA*^+^) background and 9 knock-out mutants. The mutants were *ΔrecF*, *ΔrecO,* Δ*recF-*Δ*recO,* Δ*recJ,* Δ*recJ-*Δ*recF,* Δ*recJ-*Δ*recO,* Δ*recA,* Δ*recA-*Δ*recJ,* Δ*recA-*Δ*recJ-*Δ*recO* [80,126]. The strain names are listed in S1 Table in the Supporting information. To minimise the number of active replication forks present in each cell, we cultured the cells in M9 minimal medium, supplemented with 50 µg/ml spectinomycin to maintain the SSB-mTur2 expressing mplasmid [127]. Prior to loading the microfluidic chip, cells were cultured in M9 for 5–6 hours with shaking at 1200 RPM at 37 °C, until OD_550nm_ reached 0.15-0.20. The cell culture was then pulled [127]into the microfluidic chip under flow, trapping cells within the microchannels.

### Fluorescence microscopy

The chip was mounted on a custom-built inverted microscope setup consisting of a Nikon Eclipse Ti-2 body, a 1.49 NA 100x objective, Sapphire LP lasers (Coherent, USA) and a 512 x 512 pixel EM-CCD camera (C9100-13, Hamamatsu, Japan). All images were recorded using the 1.5x multiplier within the microscope, resulting in a 50x50 μm field of view with a pixel size of 0.11 µm. At each time point in the timelapse series, two images were recorded: one capturing red mKate2 fluorescence for the cell detection, and another capturing blue mTur2 fluorescence from the SSB fusion. For mKate2 images, the sample was excited in epifluorescence mode with 568 nm laser light for 100 ms, using 1.2 mW of laser power. Emitted light between 610 – 680 nm (ET 645/75m filter, Chroma) was collected on an EM-CCD camera, using an EM gain value of 140 (Fig 1B). For SSB-mTur2 images, the sample was excited in epifluorescence mode with 458 nm laser light for 100 ms, using 0.2 mW of laser power. Emitted light between 468 – 495 nm (ET 485/30m filter, Chroma) was collected on an EM-CCD camera, using an EM gain value of 160 (Fig 1B). The timelapse interval was 5 minutes prior to UV irradiation. The frequency was increased to once every 3 minutes in the period after UV exposure. The positions were monitored for at least 4 hours before and after the UV treatment. A temperature-controlled stage was used (TIPA Plate Adapter for Nikon Ti2, Okolab s.r.l.) to keep the chip at 37 °C during the measurements. At each time-point, 40–50 positions were monitored, capturing 150–200 microchannels. With 4–6 cells per microchannel, approximately 1000 cells were imaged at each time point and 130,000 segmented cell images per experiment. For this study, with 3–4 repeats with each of the 10 strains, data was generated for about 4,000,000 *E.coli* cell images with their corresponding fluorescent SSB foci.

### Exposing the *E. coli* cells to UV

For the UV treatment a UV light source was used at a specific distance from the glass surface of the microfluidic chip, to reach a power of 5 J/m^2^ in the microchannels. (See S3 Fig) This distance was calibrated by measuring the UV power through a piece of PDMS with a similar thickness to the microfluidic chip used for microscopy measurements. The source was a UV-C lamp equipped with a mechanical shutter. The typical power was about 40 µW/cm^2^. After calibration, the lid of the temperature-controlled stage was removed, and the UV source was moved above the mounted chip. The UV shutter was opened for the number of seconds required to reach 5 J/m^2^. The recording started automatically and immediately after the UV exposure. After the first round of acquisitions the lid was put back, and the cells were imaged with a 3-minute interval for 4 hours at 37 °C.

### Image processing and analysis

A custom image-processing pipeline [109] was used to detect, segment, and track the bacterial cells throughout the experiment. All three tasks were performed with custom made deep-learning models, based on the concepts of YOLO for cell detection, U-net for cell segmentation, and graph neural network MOT for cell tracking [109,128–130]. The pipeline was written in Python (version 3.9) using Tensorflow (version 2.9) libraries for deep learning. The details of the pipeline, including the accuracies of the models, are described in detail in our recently published methods paper [109].

In each experiment, each time frame contained about 1000 cells distributed over about 150 microchannels. The location and contour of each cell was obtained using deep learning models. First the cell was detected, and a bounding box was assigned. Then the bounding box was used to crop the cell image. This crop was processed by the segmentation model to obtain a mask image. The contour is the periphery of the mask image. The contour was then used to extract the SSB focus data from the corresponding cell in the mTur2 image.

All the processed data of the cells and their SSB foci is available in csv format. (See Data Availability).

### The UV plots

The average focus intensity values plotted throughout the main text were calculated via the following series of steps. First, all foci were detected and measured by 2D Gaussian curve fitting. Second, a per-cell average value was calculated for all cells that contained foci. Third, a per-timepoint average was calculated by averaging all the per-cell values for each timepoint. The average number of foci per cell at each timepoint was simply calculated from lists of per-cell focus counts. Before plotting, curves were smoothed using a kernel of three data points. To better compare the different strains, which had somewhat different SSB focus intensities prior to UV treatment, the post-UV data was normalized to the average value of the pre-UV data for each experiment. The UV response is therefore presented in percentage and is relative to the pre-UV conditions. The plots showing the absolute values of each recording are presented in the supplementary information (S4 to S13 Figs).

### The weighted mean of repeats

Values from experimental repeats were combined by averaging the mean value for each repeat, weighted by the number of values available for that repeat. Before plotting the curves were smoothed using a kernel of three data points.

## Supporting information

S1 Text Text. (DOCX)

S1 Table The list of all strains used. All strains are equipped with an *ssb-mTur2* allele, which replaces the native *ssb* gene, as well as a plasmid that constitutively expresses the fluorescent protein mKate2 in the cytosol [80,126].
(DOCX)

S1 Fig A microfluidic chip with active flow in the microchannels. The chip design in an adaptation of the mother-machine concept [110,111] A) *E. coli* cells are pulled into the chip under flow, via the main channels (lower part of the illustration). The circuit is designed to generate a pressure difference across the microchannels such that an active flow loads cells into the microchannels and provides the cells with nutrients during the experiment. B) During the experiments, the first cell in each microchannels (the mother cell) divides and the offspring is pushed out of the channel. The offspring are carried out of the chip under flow, via the main channels. C) A fluorescence microscopy image of *E. coli* cells loaded in the microchannels of the PDMS chip, fabricated with a master mold produced in house.
(PNG)

S2 Fig The microchannels of the microfluidic chip. (Left): A schematic with the microchannel, the filter to trap the cells, and the dummy channel. The main channels are not shown and overlap the bottom part of the microchannels and the upper part of the dummy channels. (Centre): A brightfield microscope image of the microchannel structures on the master mold. The channels are patterned on a silicon wafer with SU8 negative photoresist. The main channels are not shown. (Right): A scanning electron micrograph of the cross sections of a filter and a microchannel.
(PNG)

S3 Fig Exposing the *E. coli* cells to UV. A) The mounted microfluidic chip with the microscope objective in place below the chip. After 4 hours of pre-UV time-lapse recording, the lid of the temperature-controlled stage is removed. B) A UV light equipped with a software-controlled shutter is moved on top of the chip. The exposure time is calibrated to ensure 5 J/m^2^ of energy reaches the *E. coli* cells trapped in the microchannels. The software opens the shutter for the UV exposure, then immediately resumes imaging. Once all positions have been imaged, the lid is put back on the stage to ensure that a temperature of 37 °C is maintained. The time-lapse acquisition runs for an additional 4 hours with images taken at 3-minute intervals.
(PNG)

S4 Fig Absolute (unaveraged) focus intensities and number of foci per cell for repair-proficient (*rec^+^*) cells, pre and post UV treatment (5 J/m^2^). All repeats are included. The shaded areas represent the standard deviations. A) The brightness (intensity) of the SSB-mTur2 foci. B) The number of SSB-mTur2 foci per cell.
(PNG)

S5 Fig Absolute (unaveraged) focus intensities and number of foci per cell for Δ*recF* cells, pre and post UV treatment (5 J/m^2^). All repeats are included. The shaded areas represent the standard deviations. A) The brightness (intensity) of the SSB-mTur2 foci. B) The number of SSB-mTur2 foci per cell.
(PNG)

S6 Fig Absolute (unaveraged) focus intensities and number of foci per cell for *ΔrecO* cells, pre and post UV treatment (5 J/m^2^). All repeats are included. The shaded areas represent the standard deviations. A) The brightness (intensity) of the SSB-mTur2 foci. B) The number of SSB-mTur2 foci per cell.
(PNG)

S7 Fig Absolute (unaveraged) focus intensities and number of foci per cell for Δ*recF* Δ*recO* cells, pre and post UV treatment (5 J/m^2^). All repeats are included. The shaded areas represent the standard deviations. A) The brightness (intensity) of the SSB-mTur2 foci. B) The number of SSB-mTur2 foci per cell.
(PNG)

S8 Fig Absolute (unaveraged) focus intensities and number of foci per cell for Δ*recJ* cells, pre and post UV treatment (5 J/m^2^). All repeats are included. The shaded areas represent the standard deviations. A) The brightness (intensity) of the SSB-mTur2 foci. B) The number of SSB-mTur2 foci per cell.
(PNG)

S9 Fig Absolute (unaveraged) focus intensities and number of foci per cell for Δ*recJ* Δ*recF* cells, pre and post UV treatment (5 J/m^2^). All repeats are included. The shaded areas represent the standard deviations. A) The brightness (intensity) of the SSB-mTur2 foci. B) The number of SSB-mTur2 foci per cell.
(PNG)

S10 Fig Absolute (unaveraged) focus intensities and number of foci per cell for Δ*recJ* Δ*recO* cells, pre and post UV treatment (5 J/m^2^). All repeats are included. The shaded areas represent the standard deviations. A) The brightness (intensity) of the SSB-mTur2 foci. B) The number of SSB-mTur2 foci per cell.
(PNG)

S11 Fig Absolute (unaveraged) focus intensities and number of foci per cell for Δ*recA* cells, pre and post UV treatment (5 J/m^2^). All repeats are included. The shaded areas represent the standard deviations. A) The brightness (intensity) of the SSB-mTur2 foci. B) The number of SSB-mTur2 foci per cell.
(PNG)

S12 Fig Absolute (unaveraged) focus intensities and number of foci per cell for Δ*recA* Δ*recO* cells, pre and post UV treatment (5 J/m^2^). All repeats are included. The shaded areas represent the standard deviations. A) The brightness (intensity) of the SSB-mTur2 foci. B) The number of SSB-mTur2 foci per cell.
(PNG)

S13 Fig Absolute (unaveraged) focus intensities and number of foci per cell for Δ*recA* Δ*recJ* Δ*recO* cells, pre and post UV treatment (5 J/m^2^). All repeats are included. The shaded areas represent the standard deviations. A) The brightness (intensity) of the SSB-mTur2 foci. B) The number of SSB-mTur2 foci per cell.
(PNG)

S14 Fig Cell cycle periods in the pre-UV treatment experiments of *rec*^+^, *ΔrecF, ΔrecO, ΔrecF-ΔrecO.* N represents the number of cell cycles extracted from the first cell in a channel of an individual experiment. The cut-off threshold of the cell-cycle periods was set to 4 frames, accepting cycles with more than 4 frames only. This was done to omit possible detection errors. (Note that the *ΔrecF* experiment 1 was conducted with a 3-minute timelapse interval. The rest was conducted with a 5-minute timelapse interval.).
(PNG)

S15 Fig Cell cycle periods in the pre-UV treatment experiments of *rec*^+^, *ΔrecJ, ΔrecJ-ΔrecF, ΔrecJ-ΔrecO.* N represents the number of cell cycles extracted from the first cell in a channel of an individual experiment. The cut-off threshold of the cell-cycle periods was set to 4 frames, accepting cycles with more than 4 frames only. This was done to omit possible detection errors.
(PNG)

S16 Fig Cell cycle periods in the pre-UV treatment experiments of *rec*^+^, *ΔrecA, ΔrecA-ΔrecO, ΔrecA-ΔrecO-ΔrecJ.* N represents the number of cell cycles extracted from the first cell in a channel of an individual experiment. The cut-off threshold of the cell-cycle periods was set to 4 frames, accepting cycles with more than 4 frames only. This was done to omit possible detection errors.
(PNG)

S17 Fig The difference in cell length at the beginning and end of the cell cycle periods in the pre-UV treatment experiments of *rec*^+^, *ΔrecF, ΔrecO, ΔrecF-ΔrecO.* N represents the number of cell cycles extracted from the first cell in a channel of an individual experiment.
(PNG)

S18 Fig The difference in cell length at the beginning and end of the cell cycle periods in the pre-UV treatment experiments of *rec*^+^, *ΔrecJ, ΔrecJ-ΔrecF, ΔrecJ-ΔrecO.* N represents the number of cell cycles extracted from the first cell in a channel of an individual experiment.
(PNG)

S19 Fig The difference in cell length at the beginning and end of the cell cycle periods in the pre-UV treatment experiments of *rec*^+^, *ΔrecA, ΔrecA-ΔrecO, ΔrecA-ΔrecO-ΔrecJ.* N represents the number of cell cycles extracted from the first cell in a channel of an individual experiment.
(PNG)

S20 Fig The average fluorescent foci value during the normalized cell cycle, in the pre-UV treatment experiments of *rec*^+^, *ΔrecF, ΔrecO, ΔrecF-ΔrecO.* N represents the number of cell cycles extracted from the first cell in a channel of an individual experiment.
(PNG)

S21 Fig The average fluorescent foci value during the normalized cell cycle, in the pre-UV treatment experiments of *rec*^+^, *ΔrecJ, ΔrecJ-ΔrecF, ΔrecJ-ΔrecO.* N represents the number of cell cycles extracted from the first cell in a channel of an individual experiment.
(PNG)

S22 Fig The average fluorescent foci value during the normalized cell cycle, in the pre-UV treatment experiments of *rec*^+^, *ΔrecA, ΔrecA-ΔrecO, ΔrecA-ΔrecO-ΔrecJ.* N represents the number of cell cycles extracted from the first cell in a channel of an individual experiment.
(PNG)

S23 Fig The average number of fluorescent foci during the normalized cell cycle, in the pre-UV treatment experiments of *rec*^+^, *ΔrecF, ΔrecO, ΔrecF-ΔrecO.* N represents the number of cell cycles extracted from the first cell in a channel of an individual experiment.
(PNG)

S24 Fig The average number of fluorescent foci during the normalized cell cycle, in the pre-UV treatment experiments of *rec*^+^, *ΔrecJ, ΔrecJ-ΔrecF, ΔrecJ-ΔrecO.* N represents the number of cell cycles extracted from the first cell in a channel of an individual experiment.
(PNG)

S25 Fig The average number of fluorescent foci during the normalized cell cycle, in the pre-UV treatment experiments of *rec*^+^, *ΔrecA, ΔrecA-ΔrecO, ΔrecA-ΔrecO-ΔrecJ.* N represents the number of cell cycles extracted from the first cell in a channel of an individual experiment.
(PNG)
