# Supplementary material for: Spatiotemporal characterization of single-stranded DNA intermediates after UV irradiation: II. Rapid growth and effects of recA and recJ
Source: PLoS Genet. 2026 May 14;22(5):e1012110. doi: 10.1371/journal.pgen.1012110 (PMC13175385; doi:10.1371/journal.pgen.1012110)
Supplement: S1 Text — (DOCX) [file pgen.1012110.s001.docx]

**Direct Visualization of Postreplication Gap Formation and Resolution in *Escherichia coli***

1. **Effects of *recA* and *recJ***

**Remy A. A. Ripandelli^1^, Elizabeth A. Wood^2^, Andrew Robinson^1^, Antoine M. van Oijen^1^, Michael M. Cox^2*^**

^1^Molecular Horizons and School of Chemistry and Molecular Bioscience, University of Wollongong, Wollongong, Australia

^2^College of Agricultural and Life Sciences, Department of Biochemistry, University of Wisconsin-Madison, Wisconsin, USA

##

## Supporting information

**The preparation of the M9 minimal medium**

*The preparation of M9 salts (5X) 1L:*

800 ml H2O was aliquoted, and the following reagents were added:

- 33.9 g Na2HPO4-7H2O
- 15 g KH2PO4
- 2.5 g NaCl
- 5 g NH4Cl

The total was stirred until dissolved. The solution was adjusted to 1000 ml with distilled H2O and was then sterilized by autoclaving.

*The preparation of M9 medium:*

1 M MgSO4 (24.6 g MgSO4.7H2O in final volume of 100 ml H2O)

1 M CaCl2 (11.1 g in final volume of 100 ml H2O)

20 % glucose (10 g glucose in final volume of 50 ml)

All three solutions were sterilized by filtering (0.2 µm). Then the final M9 minimal medium solution was obtained by:

- 200 ml of M9 salts
- 2 ml of 1M MgSO4
- 20 ml of 20 % glucose
- 100 µl of 1M CaCl2
- Adjust the total to 1000 ml of distilled H2O

###

### Supplementary Data

All csv files with the **processed single cell and foci data** can be found at:

<https://figshare.com/articles/dataset/The_single_cell_data_set_of_Spatiotemporal_Characterization_of_Single-Stranded_DNA_Intermediates_after_UV_Irradiation_II_Effects_of_recA_and_recJ_/30814145>

DOI: *10.6084/m9.figshare.30814145*

These csv files contain the image processed data of the detections and analysis of the fluorescent *E. coli* cells in the "mother-machine" channels of the microfluidic chip. Each file represents one time lapse movie of a strain before or after exposure to UV light. Each strain has its own sub-folder containing multiple csv files, one for each repeat. The exact strain information can be found in S1 Table.

A subset of the **time lapse tiff files** used to obtain the processed single cell data can be found at:

<https://doi.org/10.6084/m9.figshare.30814493>

DOI : *10.6084/m9.figshare.30814493*

This dataset provides a representative subset of the raw fluorescence microscopy data used to study postreplication gap formation and repair in *E****.*** *coli* following UV irradiation at 5 J/m². Because the full dataset comprises hundreds of gigabytes of time-lapse microscopy files, this Figshare deposit includes 2–3 example positions per strain. Each example corresponds to a single microfluidic chip position and contains the complete time-lapse recording from that position. For every position, both the pre-UV treatment time-lapse and its corresponding post-UV time-lapse are provided. Imaging was performed using two excitation wavelengths: 568 nm for visualizing fluorescently labeled cells and 458 nm for detecting SSB-based fluorescent gap-marker signals. The folder structure reflects the experimental layout. At the top level, the pre-UV and post-UV directories each contain subfolders for every *E. coli* strain included in the subset. Within each strain, subfolders correspond to individual microfluidic positions, each housing the full time series of:

- 568 nm channel TIFF files (cell visualization),
- 458 nm channel TIFF files (SSB gap-marker fluorescence), and
- CSV files containing automated foci-detection results for the corresponding 458 nm frames.

Note that these tiff files are already corrected for the laser beam profile.

### Supplementary Text: The calculation of the curves

**The cell cycle plots**

The image analysis script detects, segments and tracks the cells in a time lapse recording. With this information full cell cycles are extracted from the recording. These cycles vary in number of frames. The normalized cell cycle is constructed with a chosen number of virtual frames. In this case 100 virtual frames are used. These frames are called virtual because the values in the real cycle frames are projected (interpolated) onto these. So, the virtual values of these virtual frames are interpolated from the actual values of a cell cycle. Therefore, a number of virtual frames is chosen that is always higher than the actual number of frames of all cycles. This results in a projection of each cycle on 100 virtual frames. Now the frame-mean of all cycles can be obtained by taking the mean and standard deviation of all interpolated values belonging to the same frame.

For the plots showing the number and brightness of SSB foci, the zeros in the interpolated values are ignored. These zeros come from cycle frames in which none of the cells showed any foci. The cell cycle plots presented in this work were constructed using the tracking of the first cell in the microfluidic channel only. The cycle data from these cells are the clearest, because these are the cells trapped at the filter of the channel and are not pushed away by the offspring.

**The UV plots**

The UV plots show the average of the SSB foci data of all cells in each time lapse frame. In each frame cells are detected and segmented. The segmentations are used to extract the SSB foci data of each cell. The SSB foci data in each cell is averaged to get the mean foci data per cell. All these average foci values per cell are accumulated. Then the mean and standard deviation of all cells is taken for that frame. The average foci brightness values per frame is calculated by averaging the mean cell values of cells that contained foci only. The average number of foci per frame was calculated by averaging the mean cell values of all cells. Before plotting the curves were smoothened using a kernel of three data point. To better compare the strains, the post-UV data was normalized with the average value of the pre-UV data. The UV response is therefore presented in percentage and is relative to the pre-UV conditions. The plots showing the absolute values of each recording are presented in the supplementary information. (S4 to S13 Figs)

**The weighted mean of repeats**

The average of repeats is obtained by taking the average of the mean of the recordings in each frame. This average value is weighted with the number of values found in that frame in each recording-repeat. For example, the repeat average cell value in frame one could have been based on 1000, 1500, and 2000 cells for repeats 1, 2, and 3 respectively. The average cell values are then averaged with these number of cells as weights. Before plotting the curves were smoothened using a kernel of three data points.
