# Supplementary material for: Spatiotemporal characterization of single-stranded DNA intermediates after UV irradiation: II. Rapid growth and effects of recA and recJ
Source: PLoS Genet. 2026 May 14;22(5):e1012110. doi: 10.1371/journal.pgen.1012110 (PMC13175385; doi:10.1371/journal.pgen.1012110)
Supplement: S1 Table — All strains are equipped with an ssb-mTur2 allele, which replaces the native ssb gene, as well as a plasmid that constitutively expresses the fluorescent protein mKate2 in the cytosol [80,126]. (DOCX) [file pgen.1012110.s002.docx]

### The strain list

| **Strain number** | **Strain** | **Parent** | **Source** |
| --- | --- | --- | --- |
| 1 | EAW1169 (WT+ssb-mTur2) + pHG004 | MG1655 | [1, 2]  This work |
| 2 | CJH0080 (ssb-mTur2 ∆recF + pHG004) | EAW629 | [3]  This work |
| 3 | CJH0081 (ssb-mTur2 ∆recO + pHG004) | EAW114 | [4]  This work |
| 4 | CJH0082 (ssb-mTur2 ∆recF ∆recO + pHG004) | EAW668 | [4]  This work |
| 5 | EAW1463 (ssb-mTur2 𝚫recJ + pHG004) | EAW1168 | [4]  This work |
| 6 | EAW1811 (ssb-mTur2 +∆recJ ∆recF + pHG004) | EAW1463  P1 | This work |
| 7 | EAW1812 (ssb-mTur2 +∆recJ ∆recO + pHG004) | EAW1463  P1 | This work |
| 8 | EAW1502 (ssb-mTur2 + ∆recA + pHG004) | EAW1169  P1 | This work |
| 9 | EAW1857 (ssb-mTur2 + ∆recO ∆recA + pHG004) | CJH0081  P1 | This work |
| 10 | EAW1858 (ssb-mTur2 +∆recJ ∆recO ∆recA + pHG004) | EAW1812  P1 | This work |

Table S1. The list of all strains used. All strains had plasmid pHG004, which was plasmid pG353C expressing mKate2 [5]. All strains are thus resistant to spectinomycin at 50 μg/ml. Those listed with P1 are also resistant to kanamycin.

1. Cherry ME, Dubiel K, Henry C, Wood EA, Revitt-Mills SA, Keck JL, et al. Spatiotemporal Dynamics of Single-stranded DNA Intermediates in *Escherichia coli*. bioRxiv. 2023;Epub:2023/05/22. doi: <https://doi.org/10.1101/2023.05.08.539320> PubMed Central PMCID: PMC37214928.

2. Dubiel K, Henry C, Spenkelink LM, Kozlov AG, Wood EA, Jergic S, et al. Development of a single-stranded DNA-binding protein fluorescent fusion toolbox. Nucleic Acids Research. 2020;48(11):6053-67. doi: 10.1093/nar/gkaa320. PubMed PMID: WOS:000574284500024.

3. Henry C, Kaur G, Cherry ME, Henrikus SS, Bonde NJ, Sharma N, et al. RecF protein targeting to post-replication (daughter strand) gaps II: RecF interaction with replisomes. Nucleic Acids Research. 2023;51(11):5714-42. doi: 10.1093/nar/gkad310. PubMed PMID: WOS:000978634400001.

4. Sharma N, Cherry ME, Henry C, Wood EA, Robinson A, van Oijen A, et al. Spatiotemporal characterization of single-stranded DNA intermediates after UV irradiation: I. Post-replication gaps formed during slow growth. PLoS Genetics. 2026;submitted.

5. Ghodke H, Paudel BP, Lewis JS, Jergic S, Gopal K, Romero ZJ, et al. Spatial and temporal organization of RecA in the *Escherichia coli* DNA-damage response. Elife. 2019;8:e42761. doi: 10.7554/eLife.42761. PubMed PMID: WOS:000457827600001.
