# Supplementary figures and images for: Spatiotemporal characterization of single-stranded DNA intermediates after UV irradiation: II. Rapid growth and effects of recA and recJ
Source: PLoS Genet. 2026 May 14;22(5):e1012110. doi: 10.1371/journal.pgen.1012110 (PMC13175385; doi:10.1371/journal.pgen.1012110)

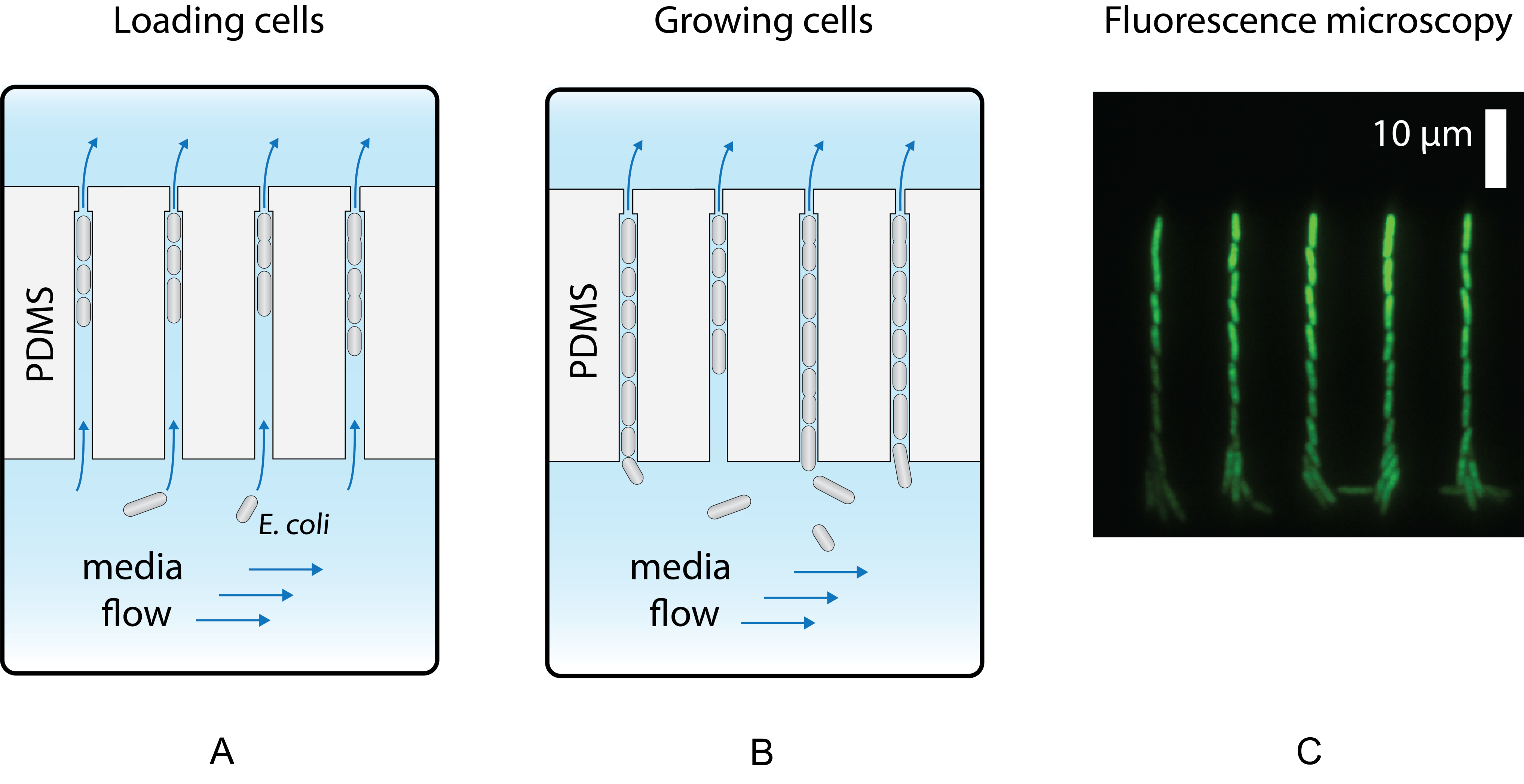

Supplement: S1 Fig — The chip design in an adaptation of the mother-machine concept [110,111] A) E. coli cells are pulled into the chip under flow, via the main channels (lower part of the illustration). The circuit is designed to generate a pressure difference across the microchannels such that an active flow loads cells into the microchannels and provides the cells with nutrients during the experiment. B) During the experiments, the first cell in each microchannels (the mother cell) divides and the offspring is pushed out of the channel. The offspring are carried out of the chip under flow, via the main channels. C) A fluorescence microscopy image of E. coli cells loaded in the microchannels of the PDMS chip, fabricated with a master mold produced in house. (PNG) [file pgen.1012110.s003.png]

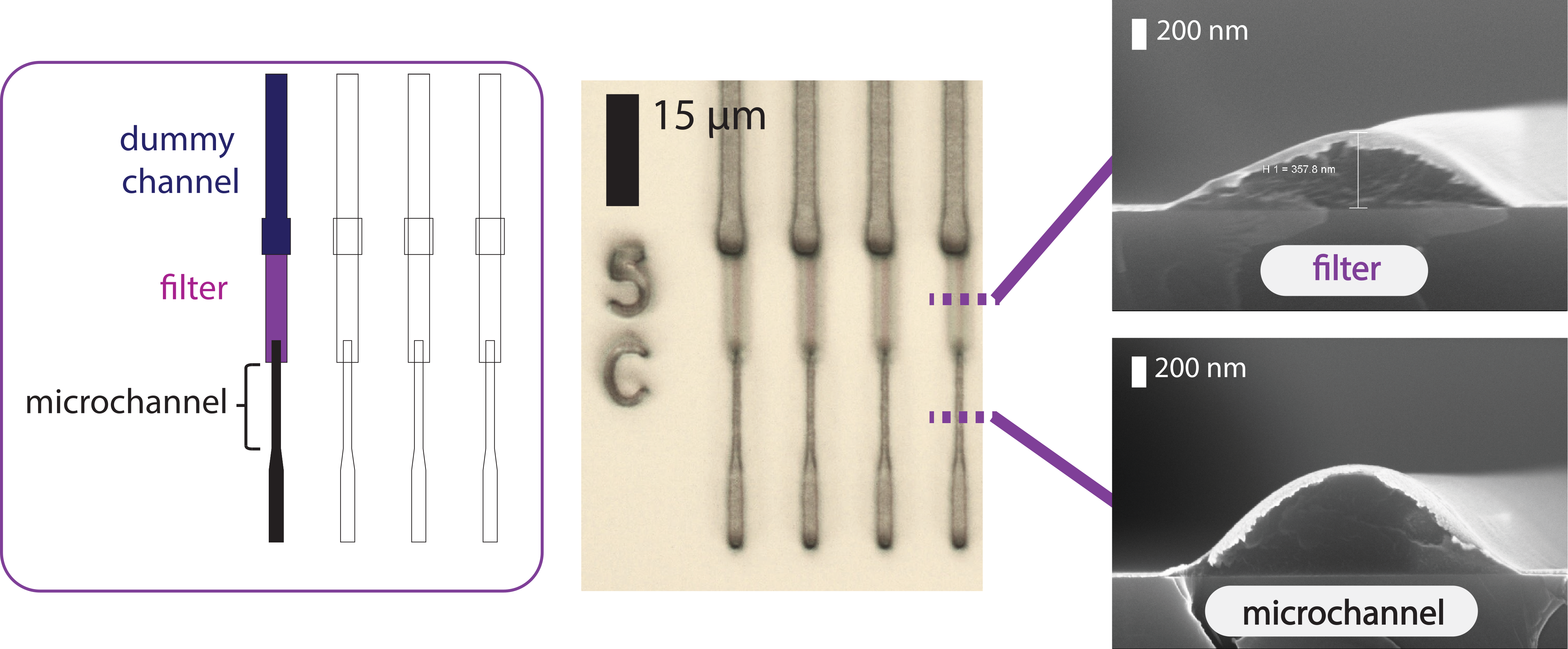

Supplement: S2 Fig — (Left): A schematic with the microchannel, the filter to trap the cells, and the dummy channel. The main channels are not shown and overlap the bottom part of the microchannels and the upper part of the dummy channels. (Centre): A brightfield microscope image of the microchannel structures on the master mold. The channels are patterned on a silicon wafer with SU8 negative photoresist. The main channels are not shown. (Right): A scanning electron micrograph of the cross sections of a filter and a microchannel. (PNG) [file pgen.1012110.s004.png]

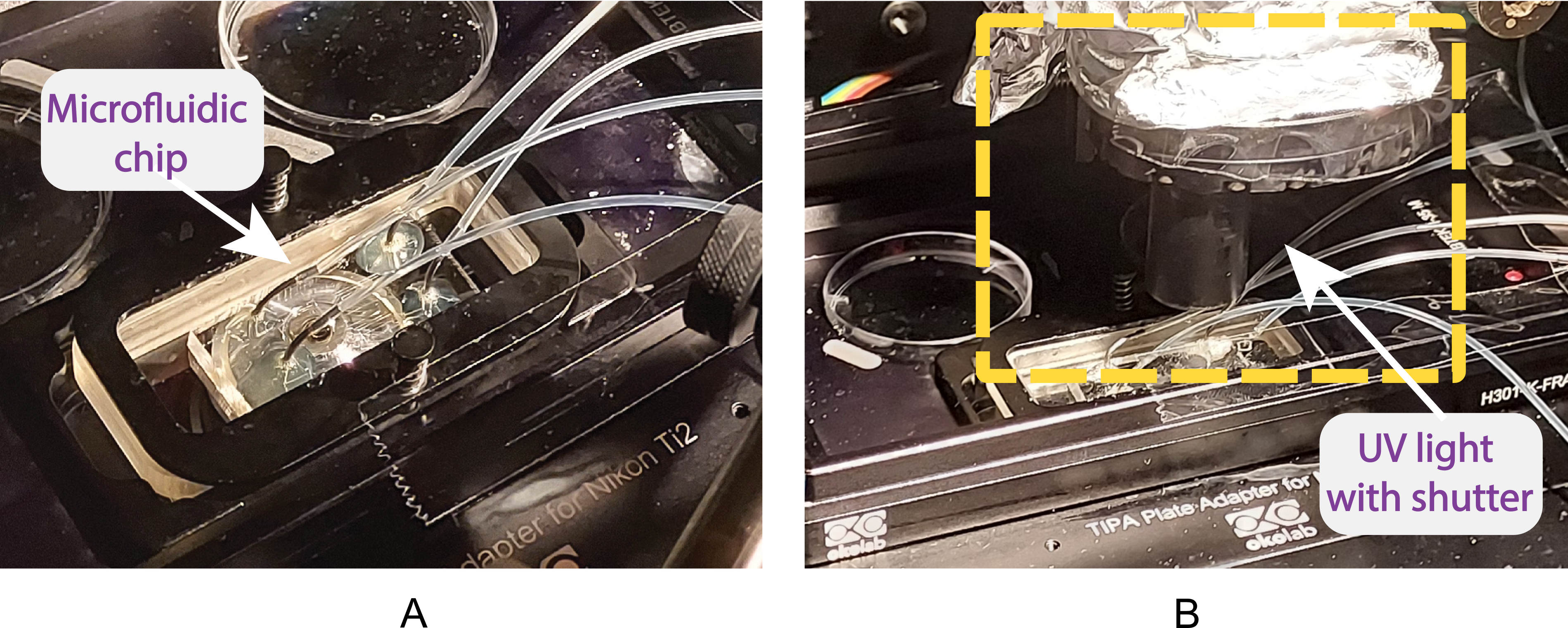

Supplement: S3 Fig — A) The mounted microfluidic chip with the microscope objective in place below the chip. After 4 hours of pre-UV time-lapse recording, the lid of the temperature-controlled stage is removed. B) A UV light equipped with a software-controlled shutter is moved on top of the chip. The exposure time is calibrated to ensure 5 J/m2 of energy reaches the E. coli cells trapped in the microchannels. The software opens the shutter for the UV exposure, then immediately resumes imaging. Once all positions have been imaged, the lid is put back on the stage to ensure that a temperature of 37 °C is maintained. The time-lapse acquisition runs for an additional 4 hours with images taken at 3-minute intervals. (PNG) [file pgen.1012110.s005.png]

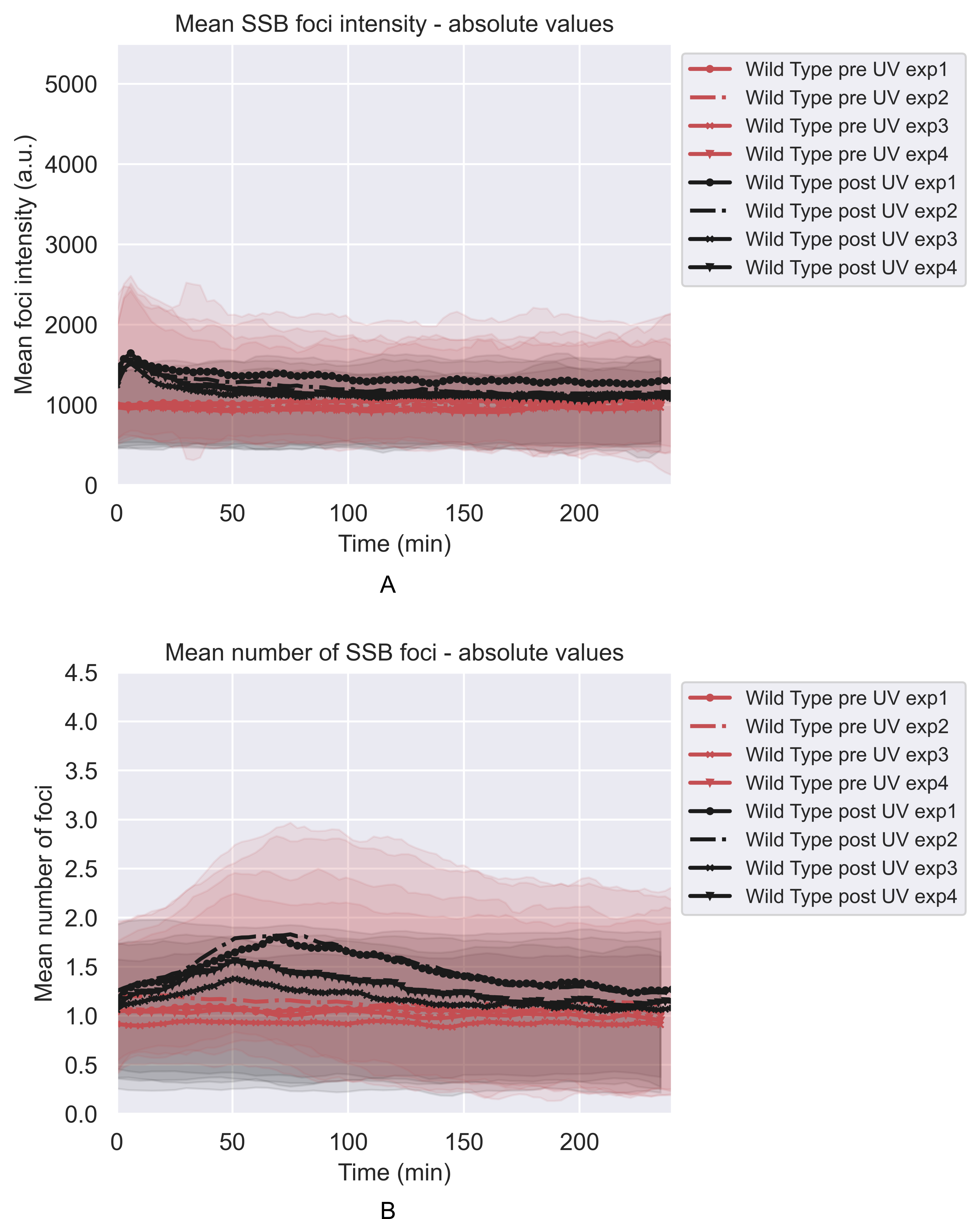

Supplement: S4 Fig — All repeats are included. The shaded areas represent the standard deviations. A) The brightness (intensity) of the SSB-mTur2 foci. B) The number of SSB-mTur2 foci per cell. (PNG) [file pgen.1012110.s006.png]

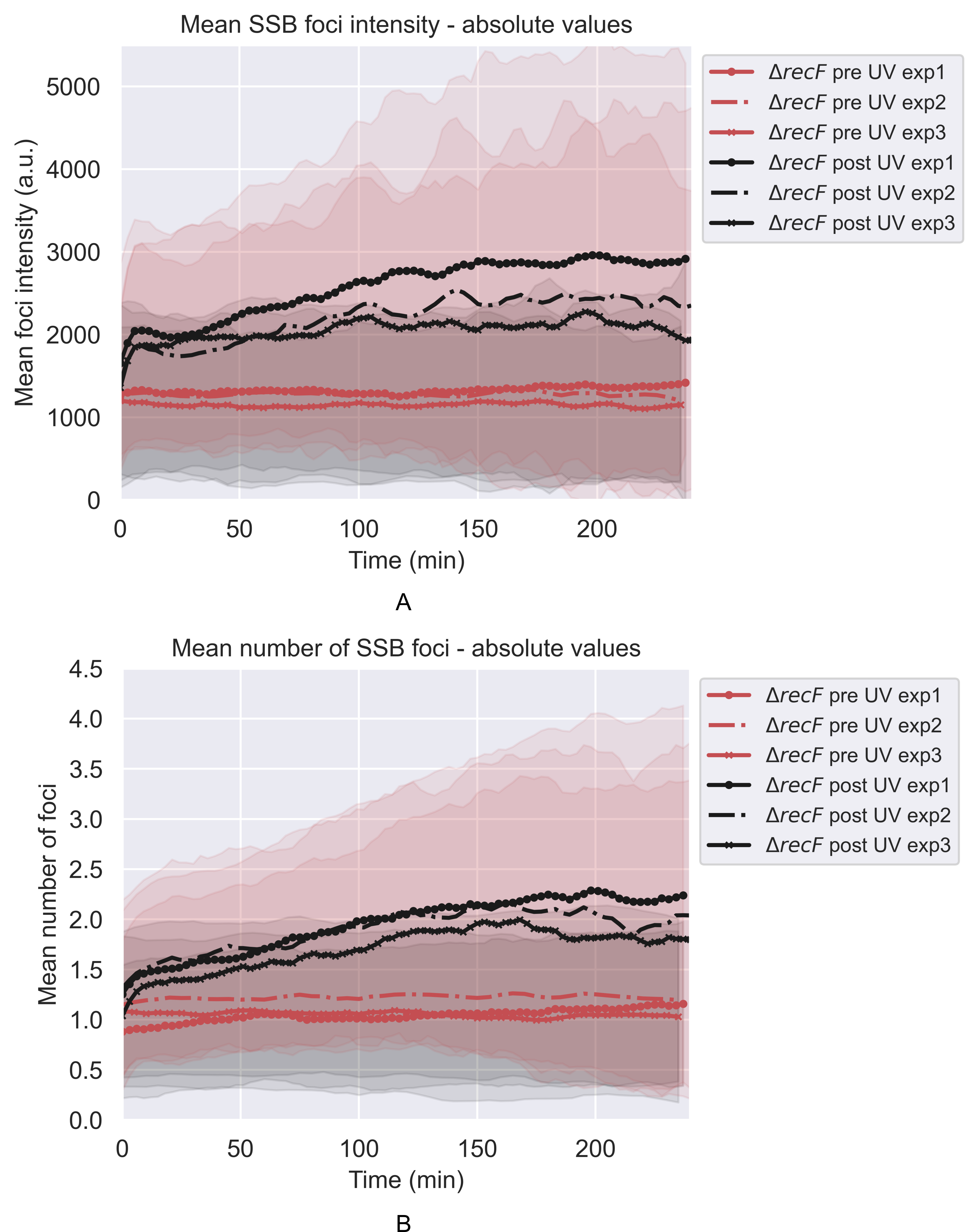

Supplement: S5 Fig — All repeats are included. The shaded areas represent the standard deviations. A) The brightness (intensity) of the SSB-mTur2 foci. B) The number of SSB-mTur2 foci per cell. (PNG) [file pgen.1012110.s007.png]

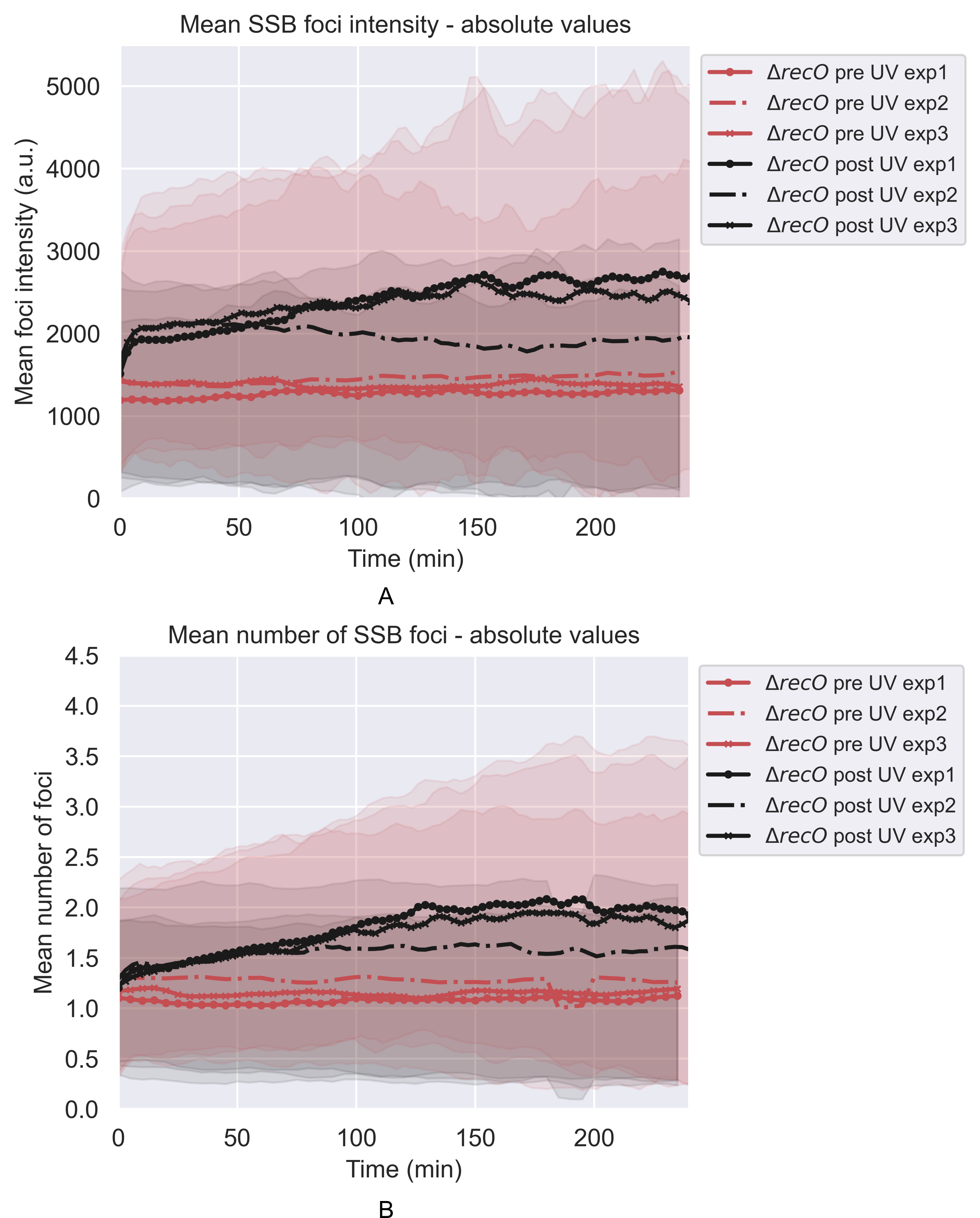

Supplement: S6 Fig — All repeats are included. The shaded areas represent the standard deviations. A) The brightness (intensity) of the SSB-mTur2 foci. B) The number of SSB-mTur2 foci per cell. (PNG) [file pgen.1012110.s008.png]

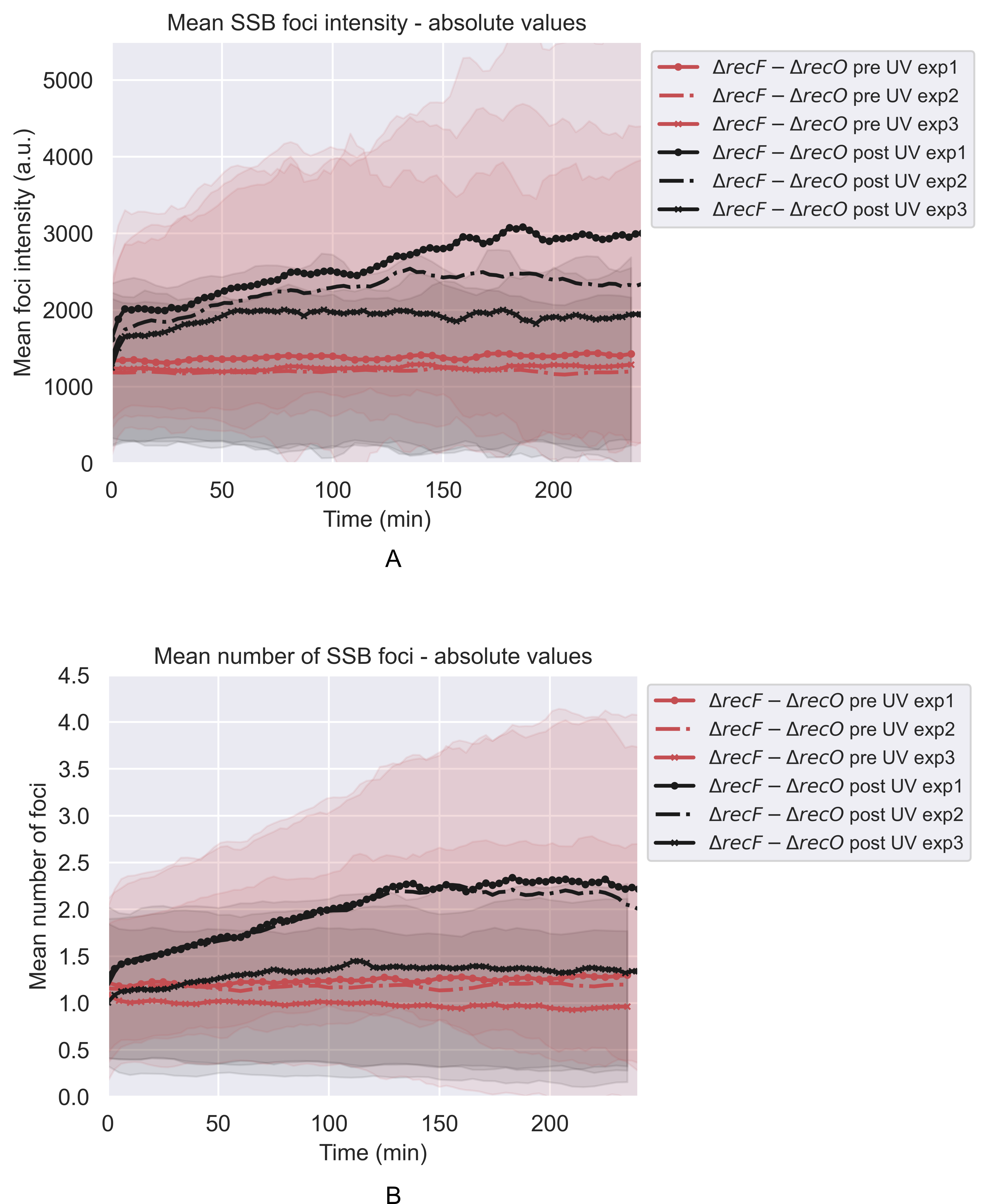

Supplement: S7 Fig — All repeats are included. The shaded areas represent the standard deviations. A) The brightness (intensity) of the SSB-mTur2 foci. B) The number of SSB-mTur2 foci per cell. (PNG) [file pgen.1012110.s009.png]

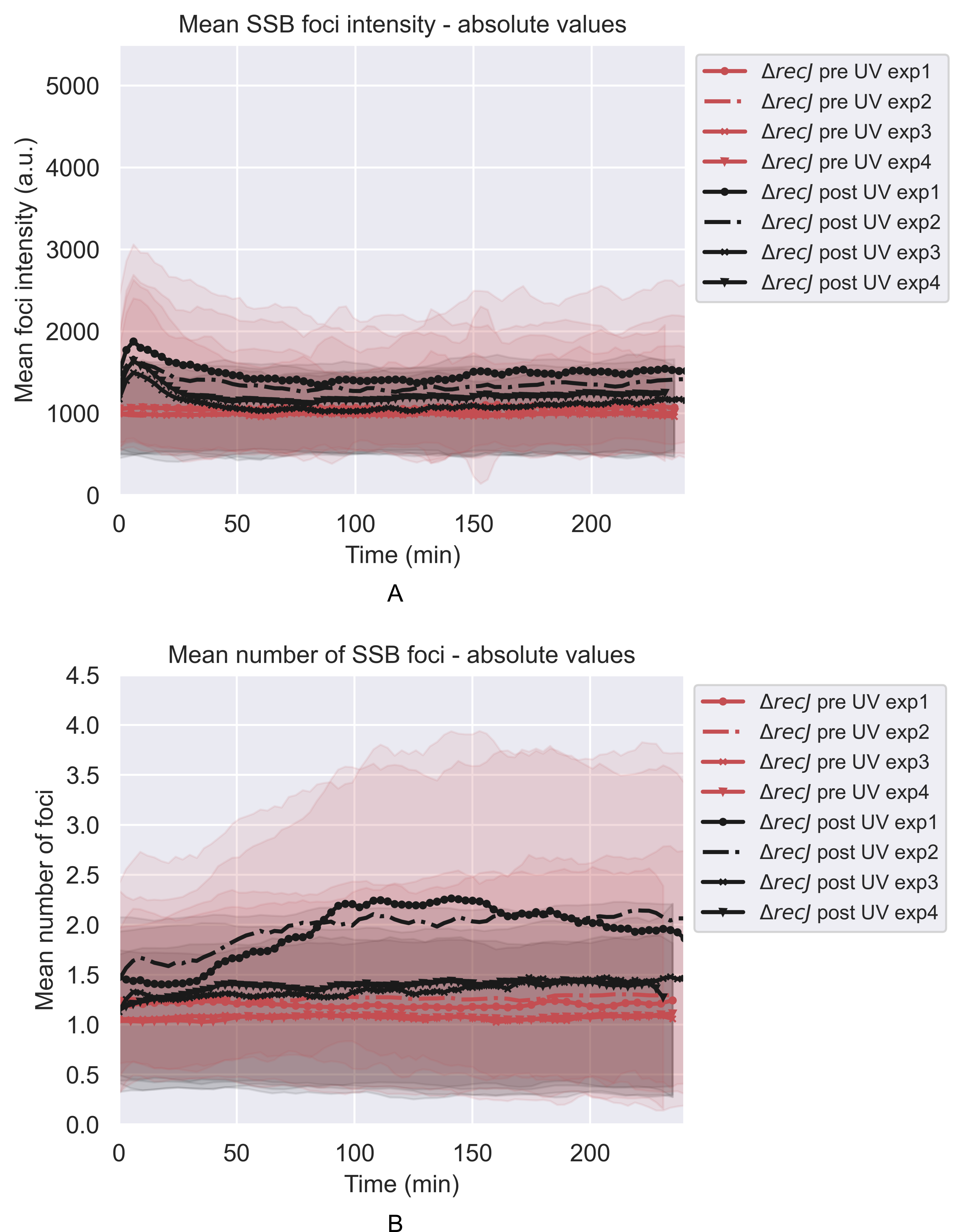

Supplement: S8 Fig — All repeats are included. The shaded areas represent the standard deviations. A) The brightness (intensity) of the SSB-mTur2 foci. B) The number of SSB-mTur2 foci per cell. (PNG) [file pgen.1012110.s010.png]

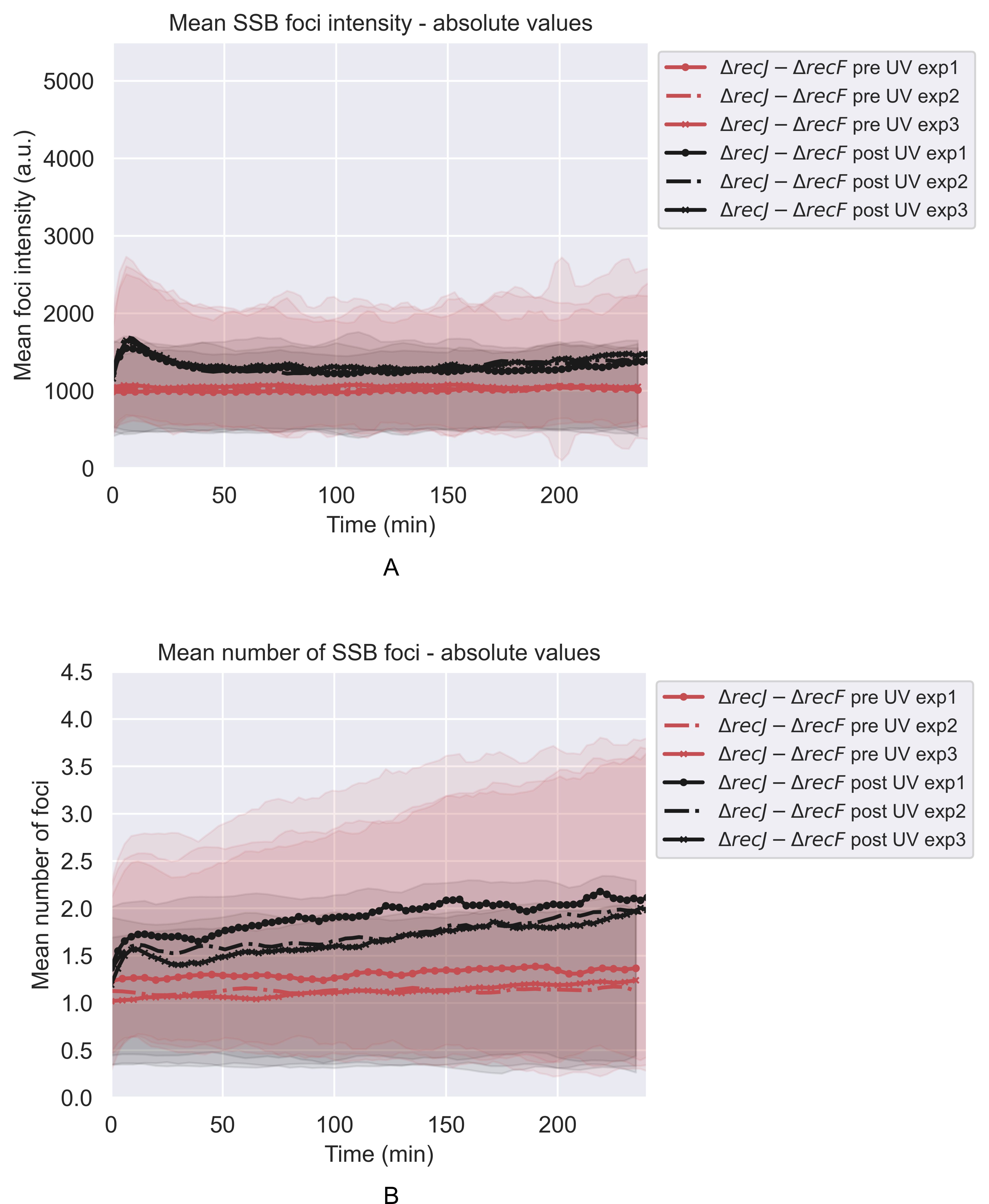

Supplement: S9 Fig — All repeats are included. The shaded areas represent the standard deviations. A) The brightness (intensity) of the SSB-mTur2 foci. B) The number of SSB-mTur2 foci per cell. (PNG) [file pgen.1012110.s011.png]

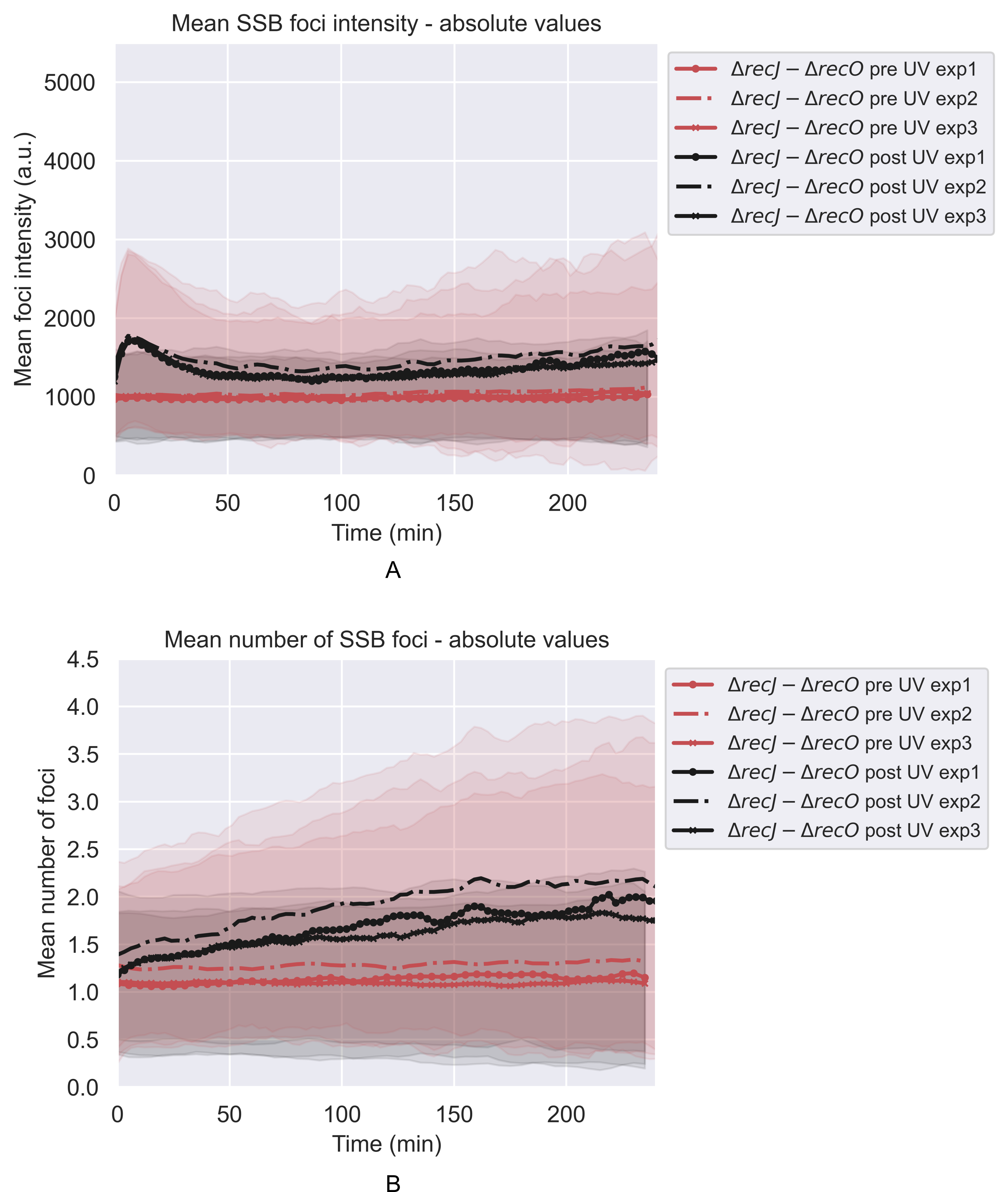

Supplement: S10 Fig — All repeats are included. The shaded areas represent the standard deviations. A) The brightness (intensity) of the SSB-mTur2 foci. B) The number of SSB-mTur2 foci per cell. (PNG) [file pgen.1012110.s012.png]

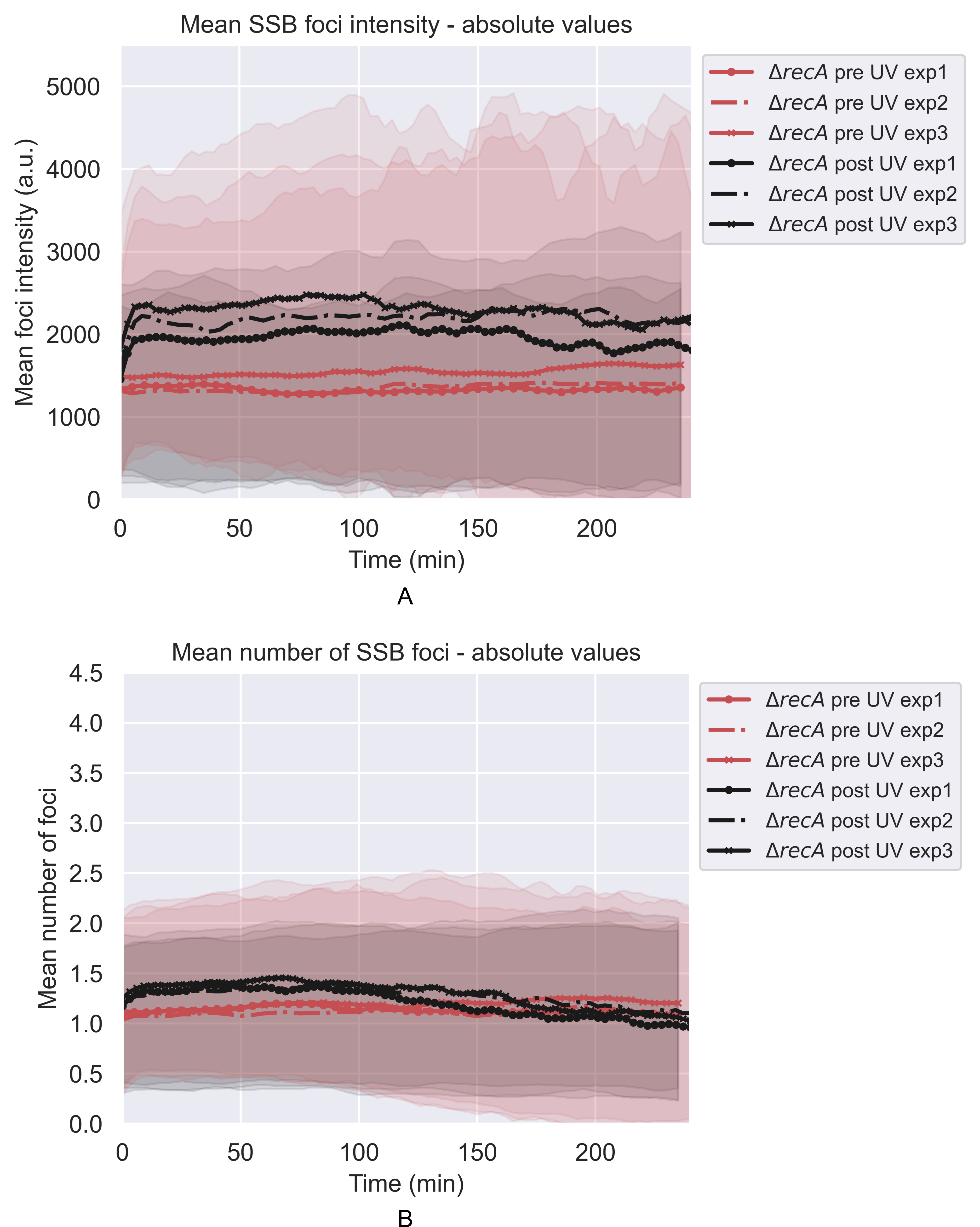

Supplement: S11 Fig — All repeats are included. The shaded areas represent the standard deviations. A) The brightness (intensity) of the SSB-mTur2 foci. B) The number of SSB-mTur2 foci per cell. (PNG) [file pgen.1012110.s013.png]

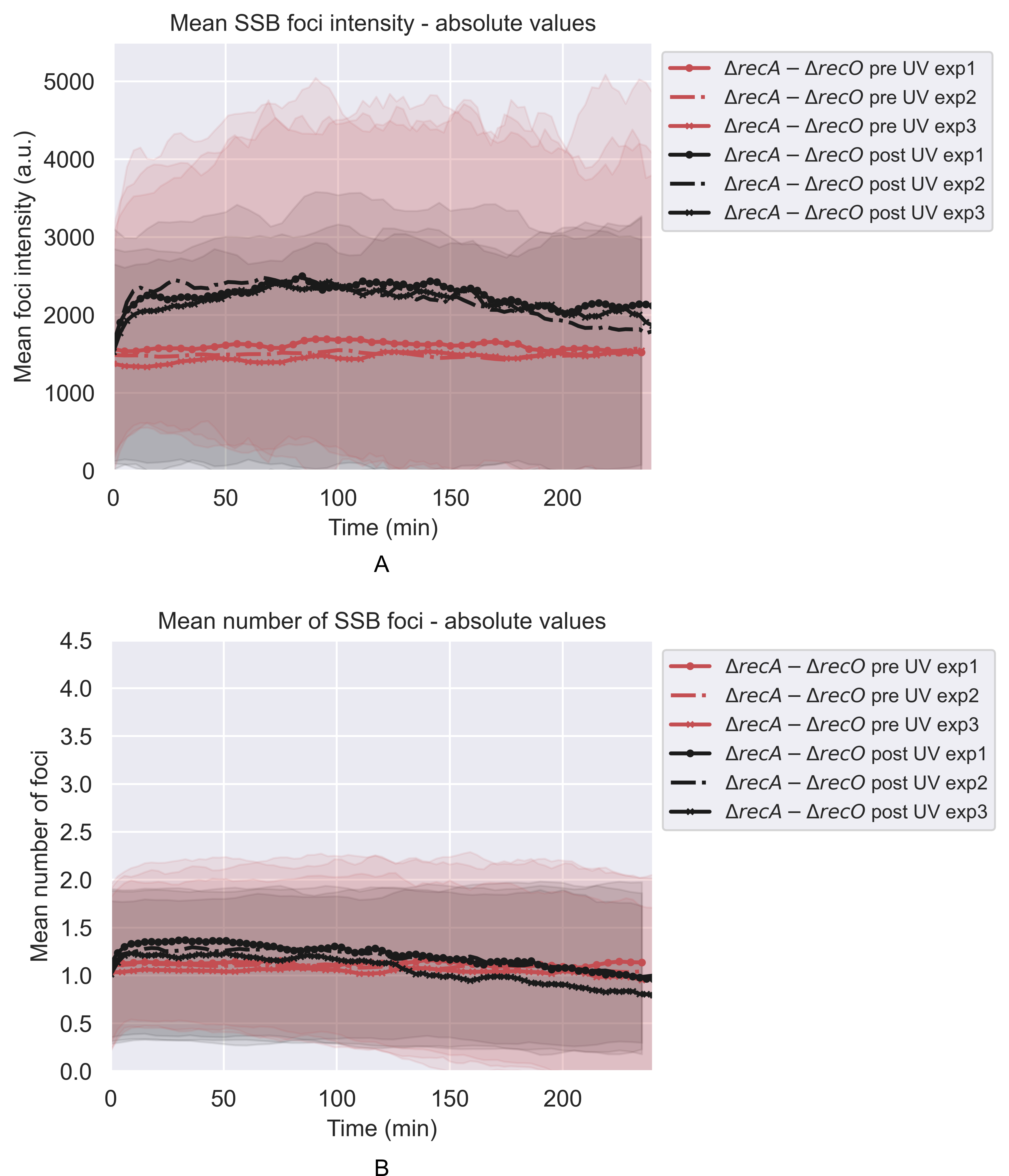

Supplement: S12 Fig — All repeats are included. The shaded areas represent the standard deviations. A) The brightness (intensity) of the SSB-mTur2 foci. B) The number of SSB-mTur2 foci per cell. (PNG) [file pgen.1012110.s014.png]

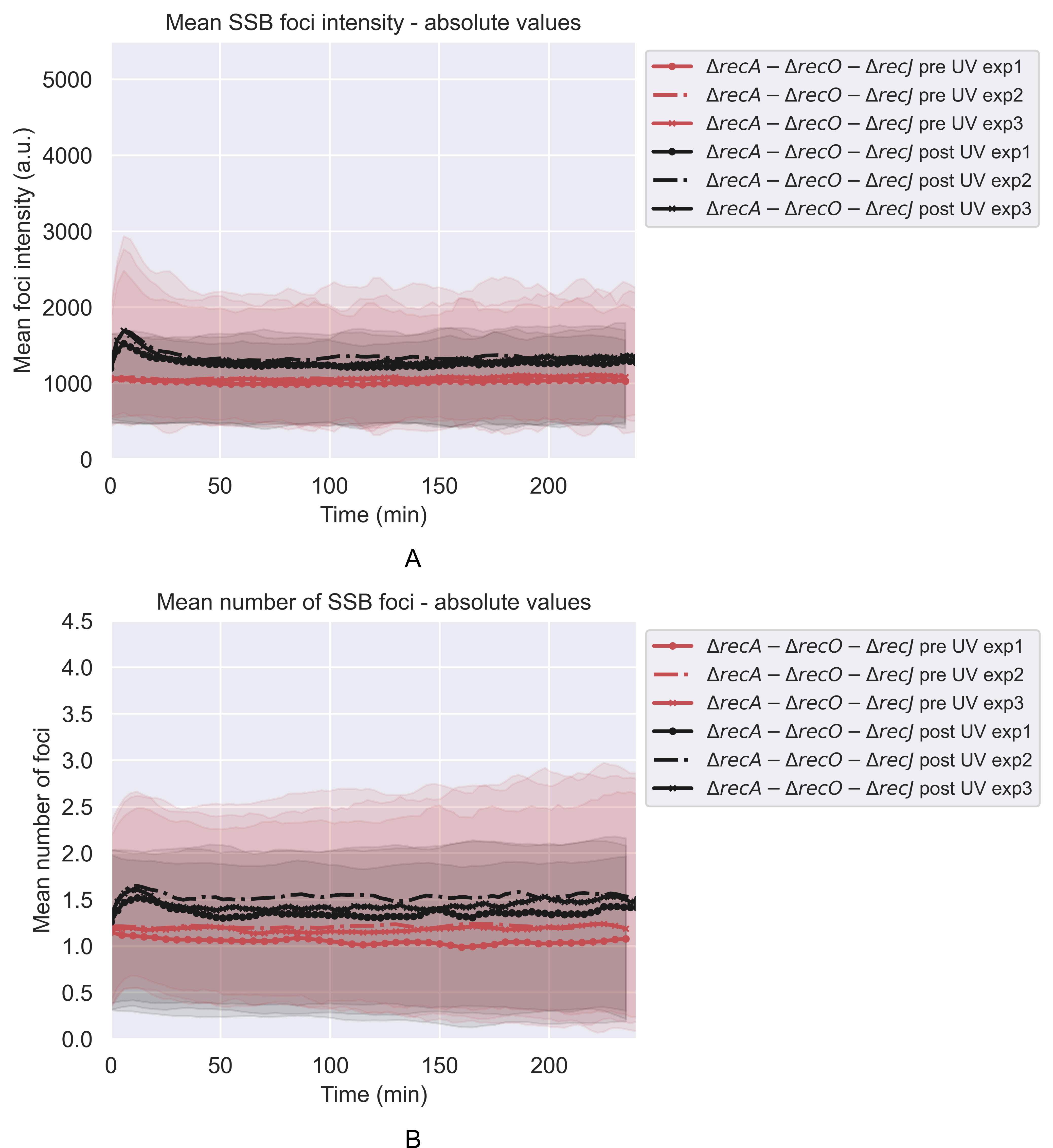

Supplement: S13 Fig — All repeats are included. The shaded areas represent the standard deviations. A) The brightness (intensity) of the SSB-mTur2 foci. B) The number of SSB-mTur2 foci per cell. (PNG) [file pgen.1012110.s015.png]

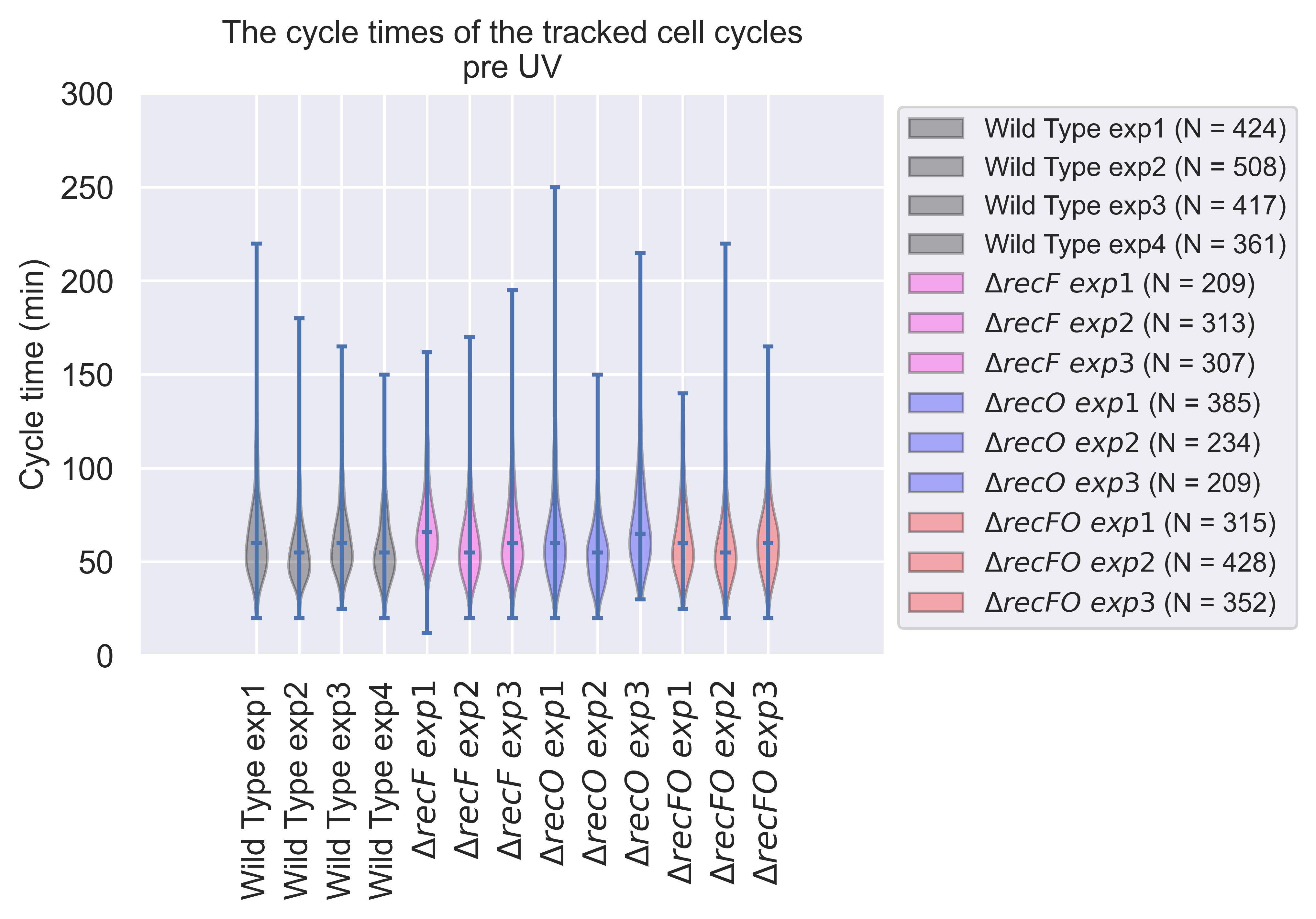

Supplement: S14 Fig — N represents the number of cell cycles extracted from the first cell in a channel of an individual experiment. The cut-off threshold of the cell-cycle periods was set to 4 frames, accepting cycles with more than 4 frames only. This was done to omit possible detection errors. (Note that the ΔrecF experiment 1 was conducted with a 3-minute timelapse interval. The rest was conducted with a 5-minute timelapse interval.). (PNG) [file pgen.1012110.s016.png]

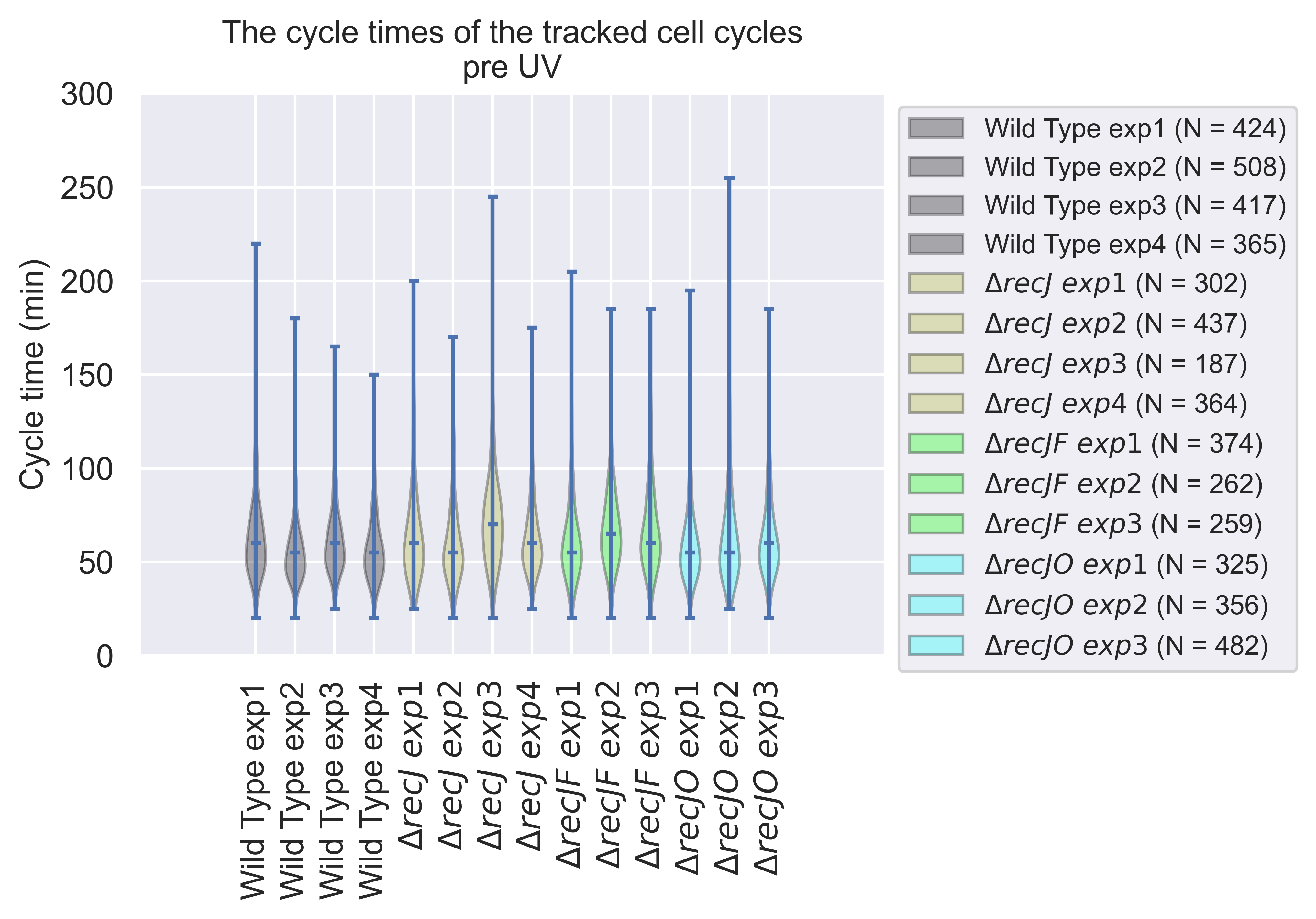

Supplement: S15 Fig — N represents the number of cell cycles extracted from the first cell in a channel of an individual experiment. The cut-off threshold of the cell-cycle periods was set to 4 frames, accepting cycles with more than 4 frames only. This was done to omit possible detection errors. (PNG) [file pgen.1012110.s017.png]

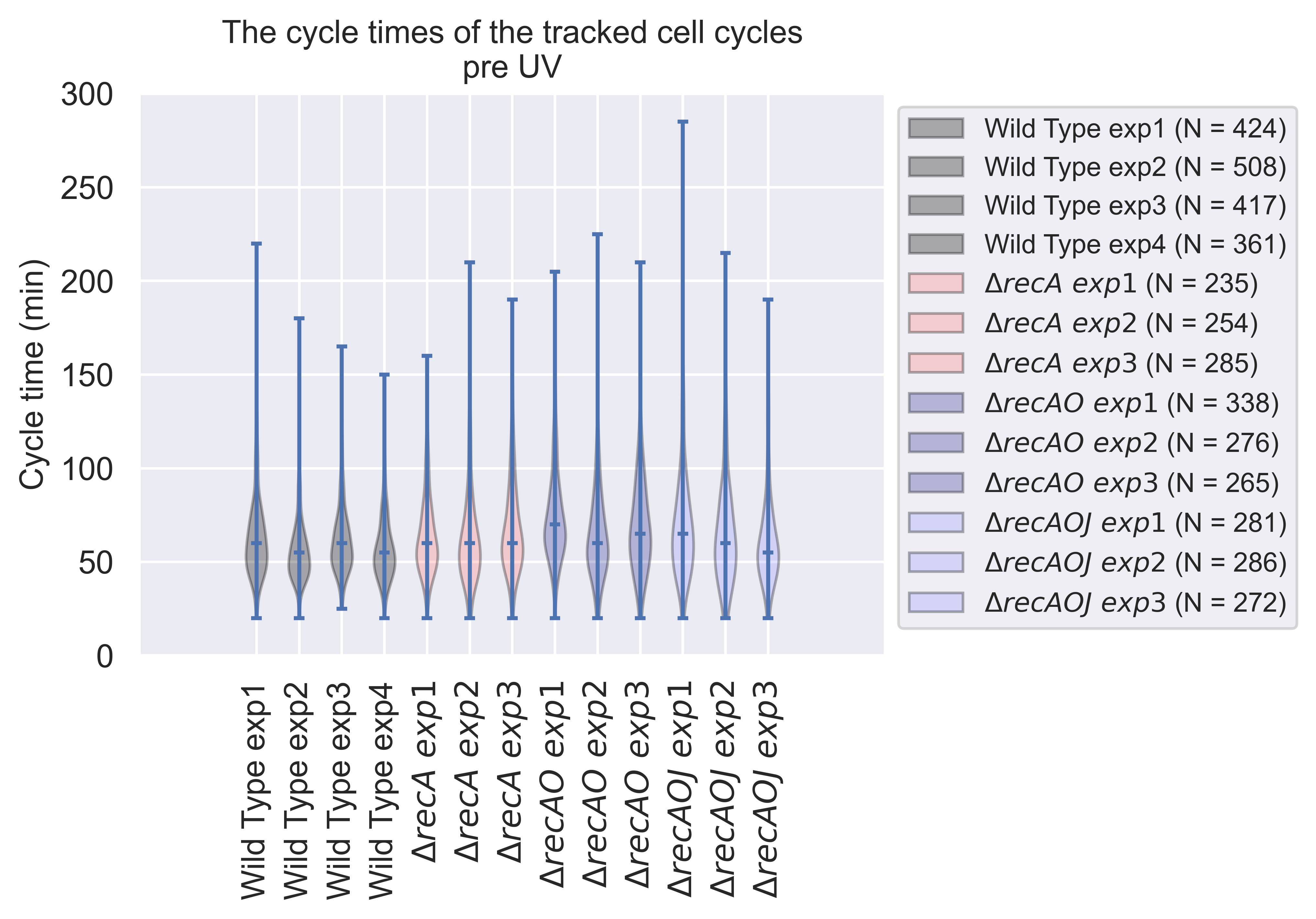

Supplement: S16 Fig — N represents the number of cell cycles extracted from the first cell in a channel of an individual experiment. The cut-off threshold of the cell-cycle periods was set to 4 frames, accepting cycles with more than 4 frames only. This was done to omit possible detection errors. (PNG) [file pgen.1012110.s018.png]

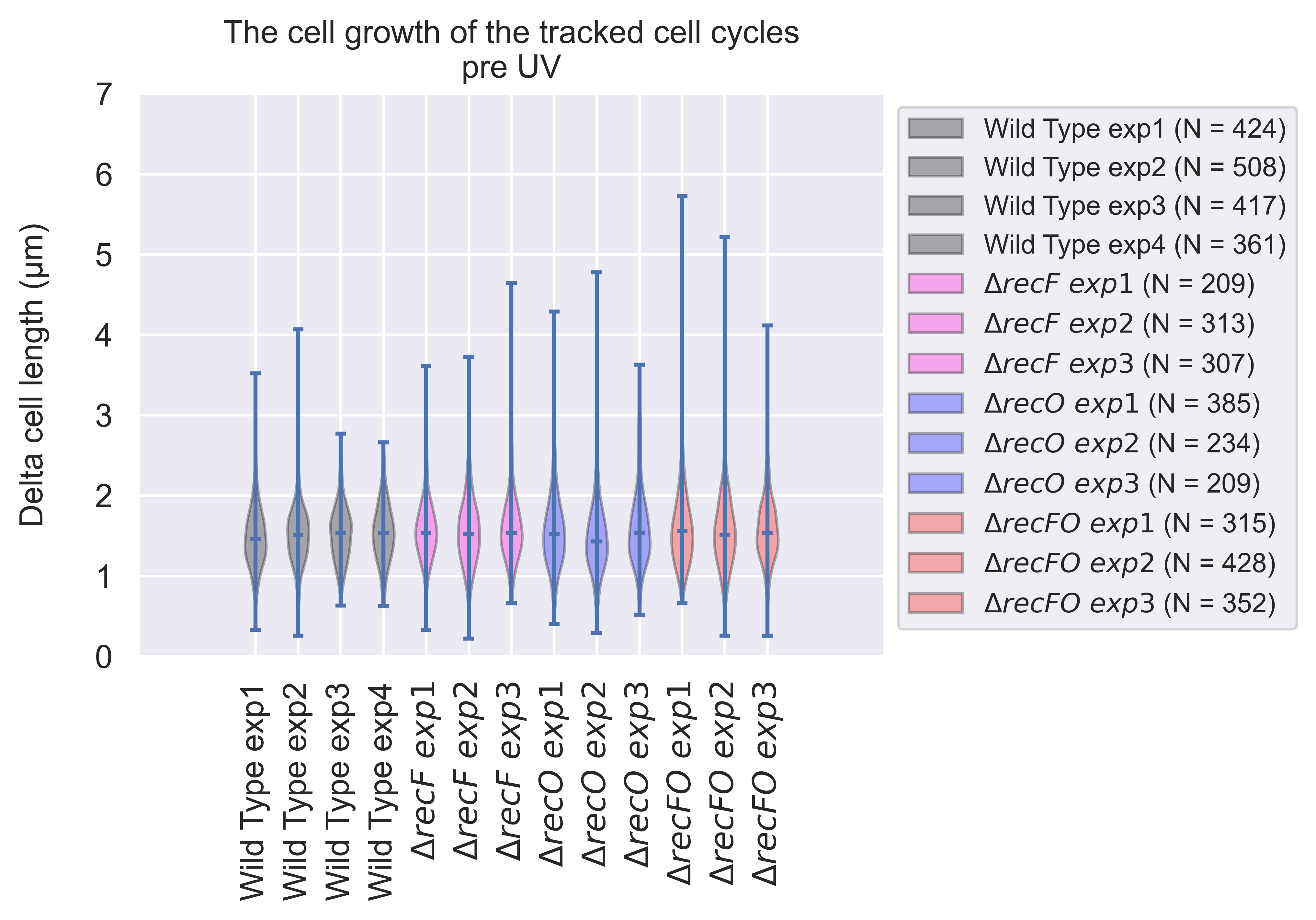

Supplement: S17 Fig — N represents the number of cell cycles extracted from the first cell in a channel of an individual experiment. (PNG) [file pgen.1012110.s019.png]

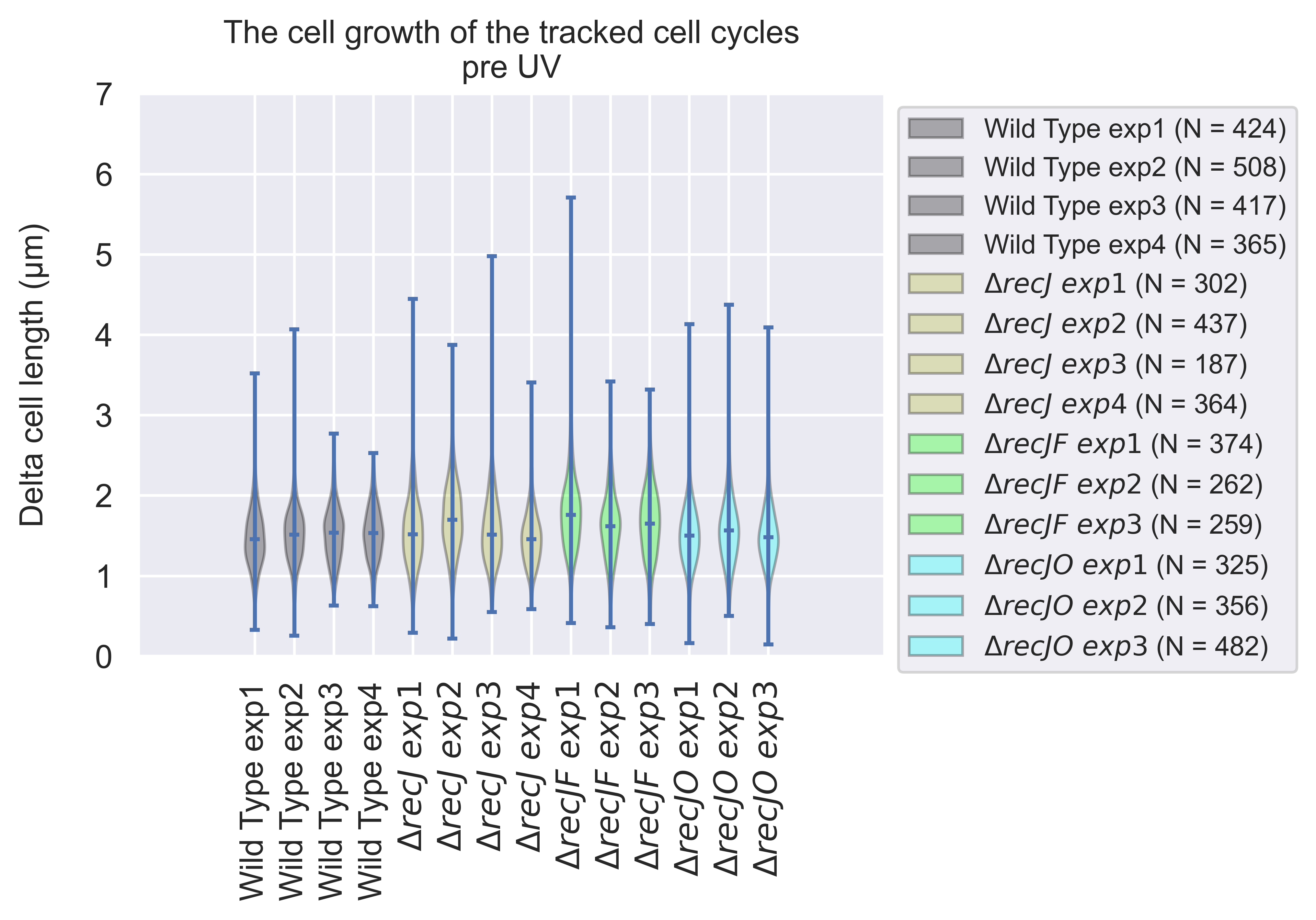

Supplement: S18 Fig — N represents the number of cell cycles extracted from the first cell in a channel of an individual experiment. (PNG) [file pgen.1012110.s020.png]

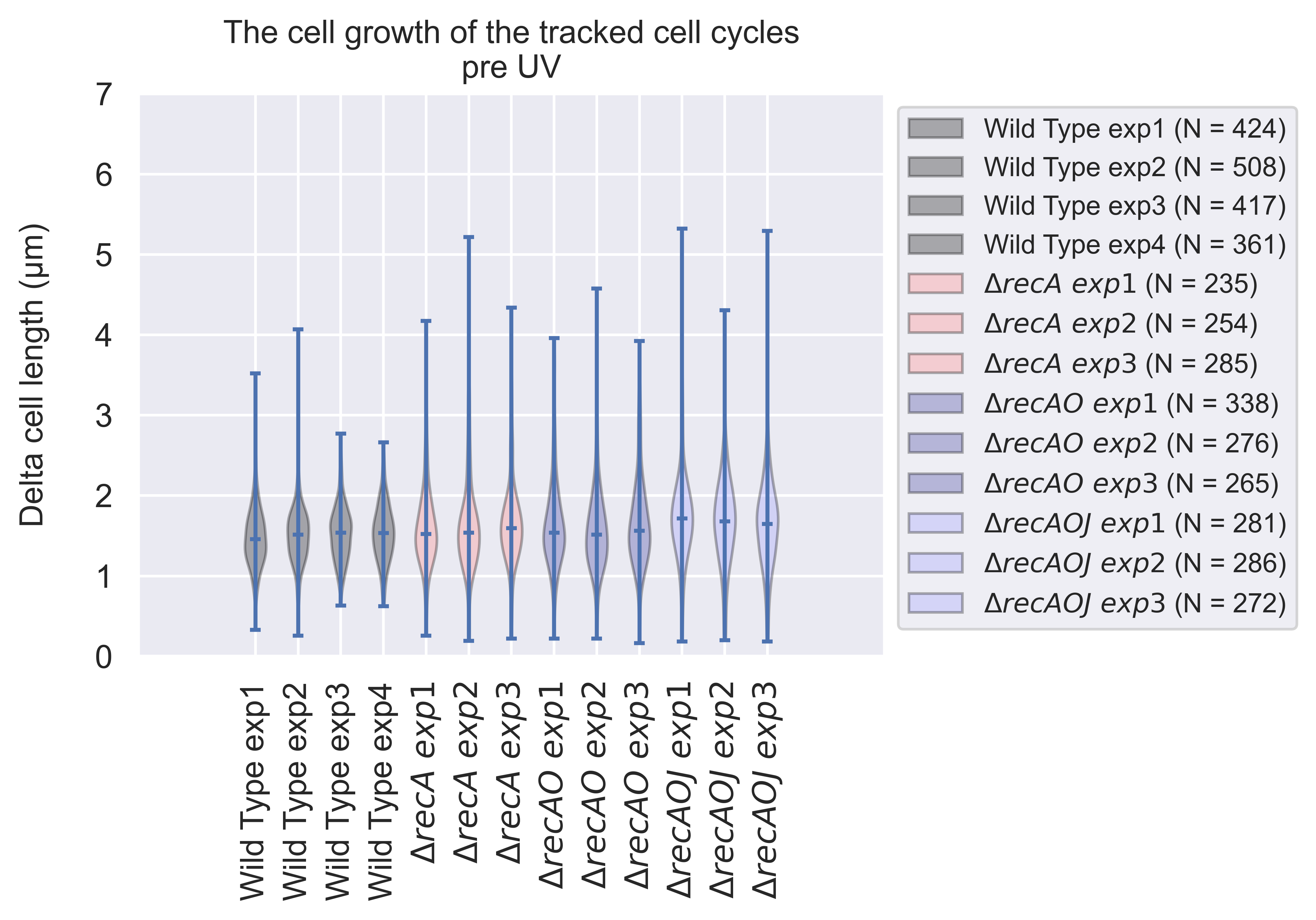

Supplement: S19 Fig — N represents the number of cell cycles extracted from the first cell in a channel of an individual experiment. (PNG) [file pgen.1012110.s021.png]

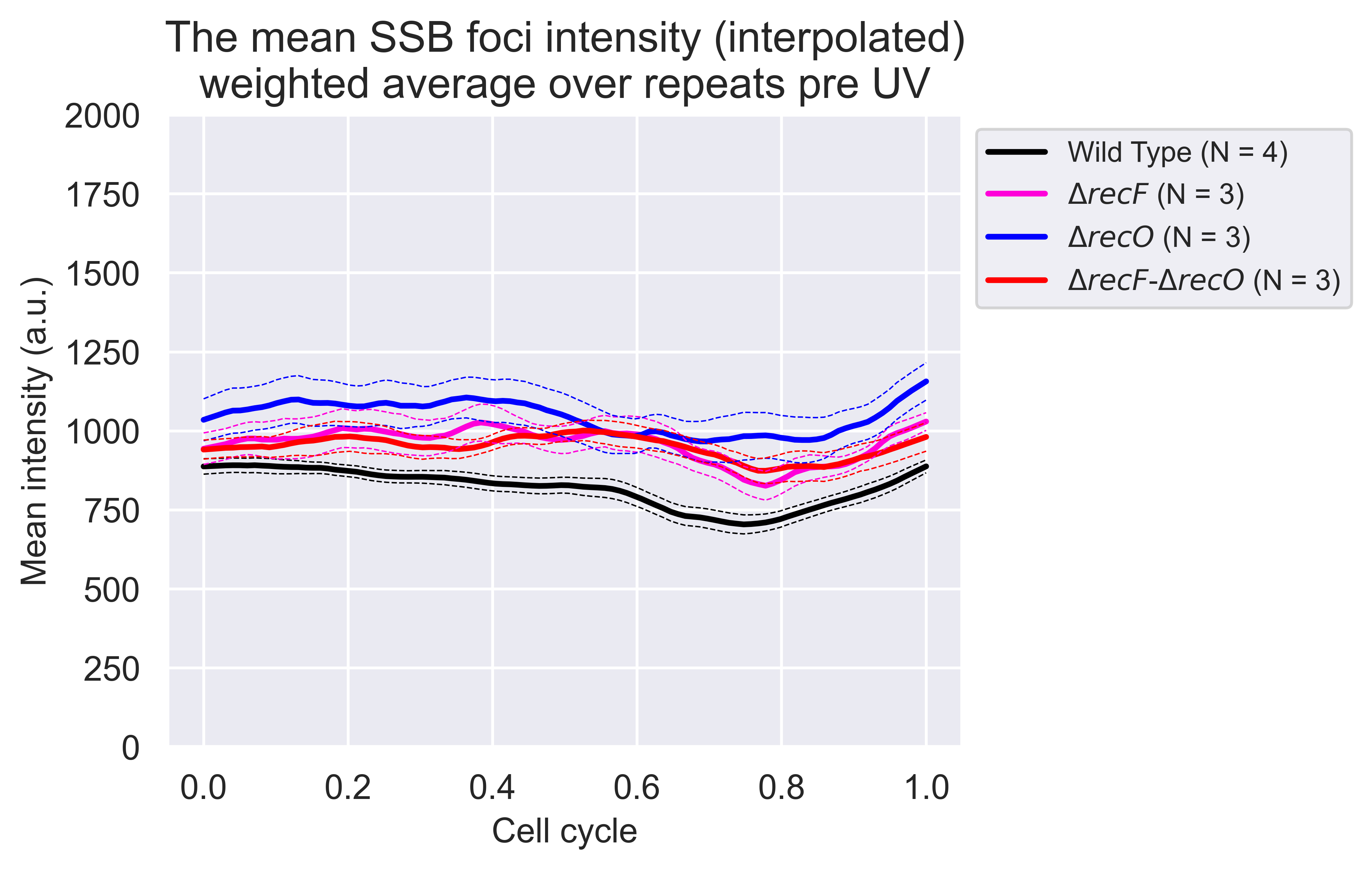

Supplement: S20 Fig — N represents the number of cell cycles extracted from the first cell in a channel of an individual experiment. (PNG) [file pgen.1012110.s022.png]

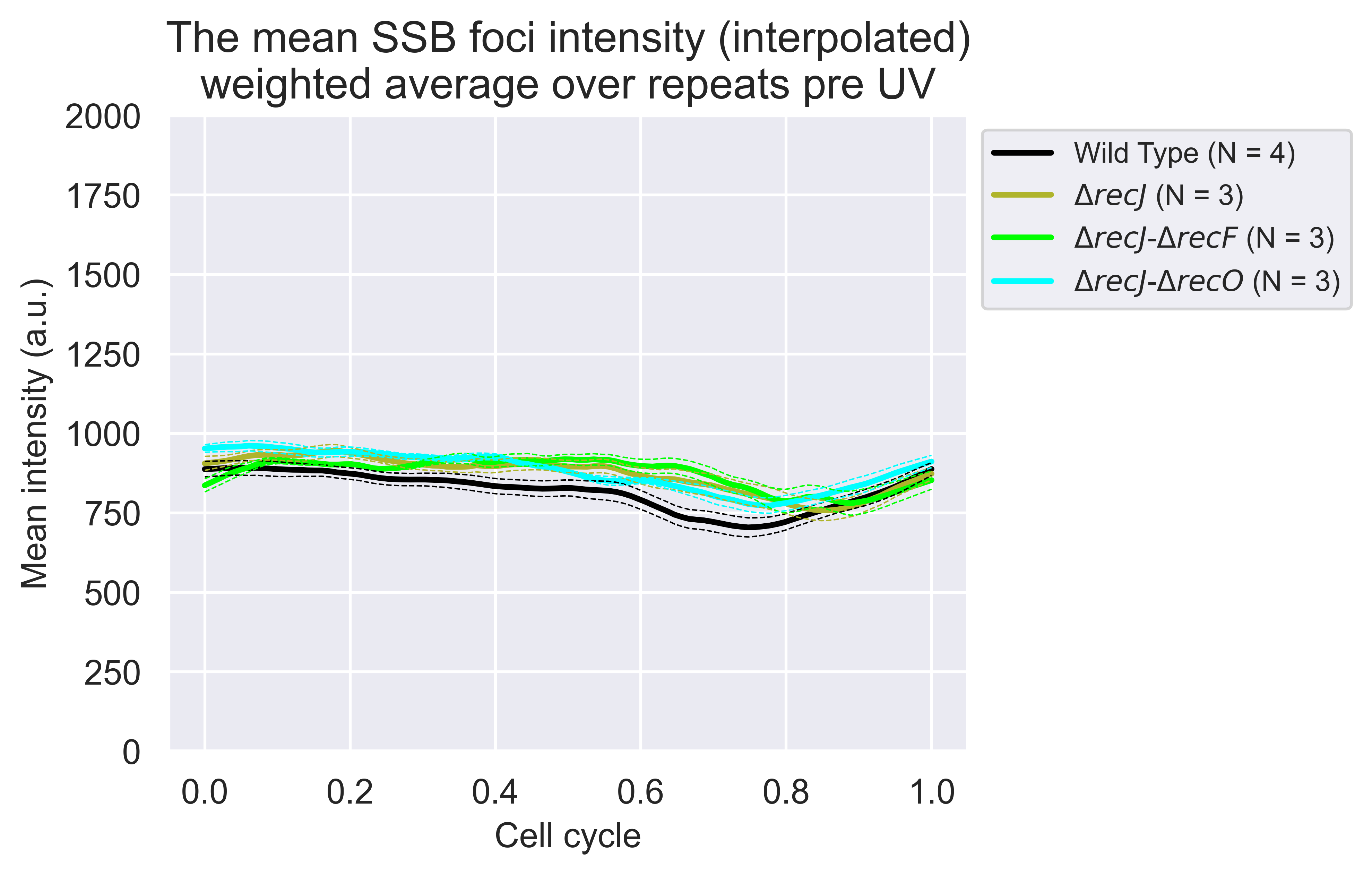

Supplement: S21 Fig — N represents the number of cell cycles extracted from the first cell in a channel of an individual experiment. (PNG) [file pgen.1012110.s023.png]

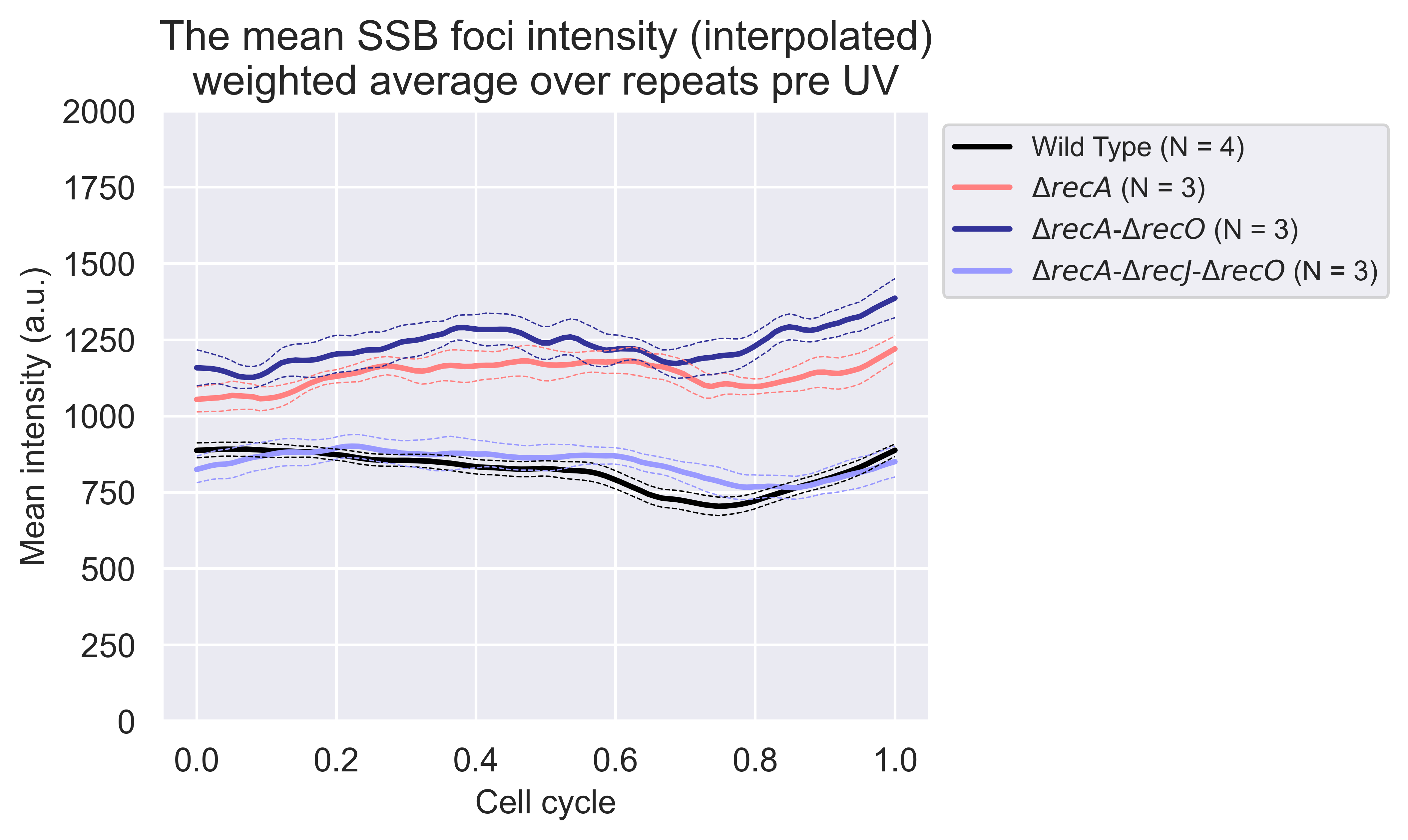

Supplement: S22 Fig — N represents the number of cell cycles extracted from the first cell in a channel of an individual experiment. (PNG) [file pgen.1012110.s024.png]

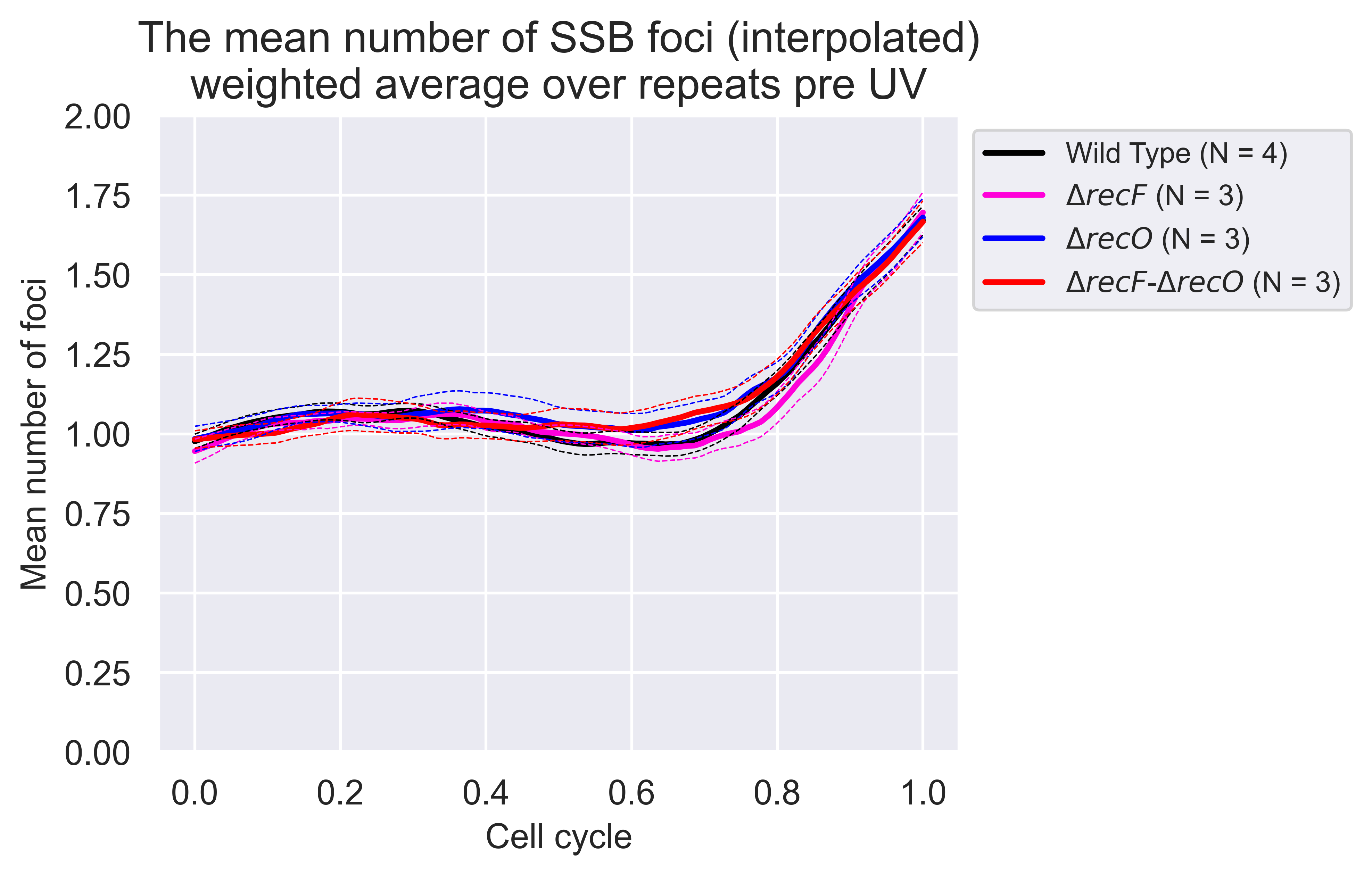

Supplement: S23 Fig — N represents the number of cell cycles extracted from the first cell in a channel of an individual experiment. (PNG) [file pgen.1012110.s025.png]

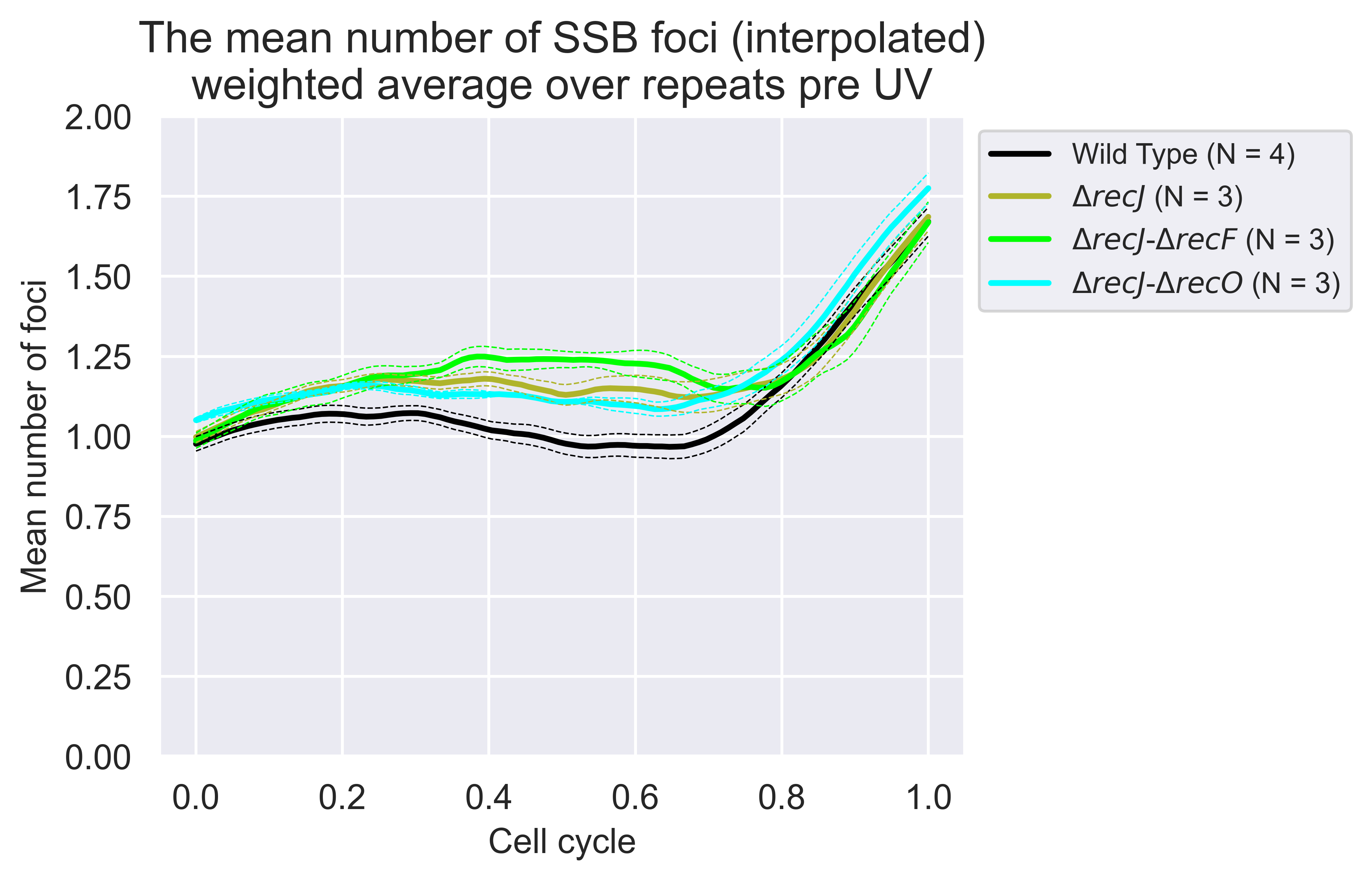

Supplement: S24 Fig — N represents the number of cell cycles extracted from the first cell in a channel of an individual experiment. (PNG) [file pgen.1012110.s026.png]

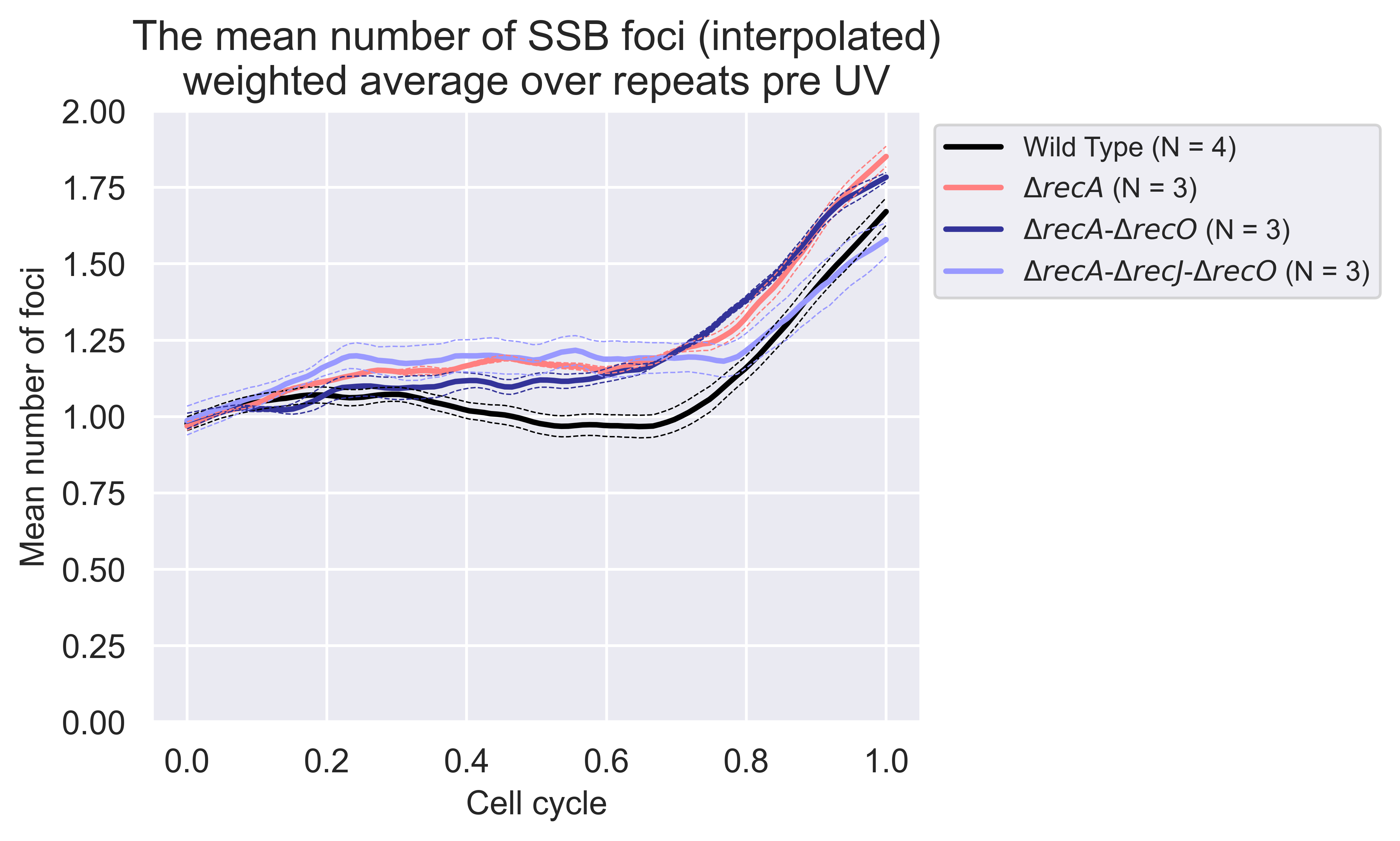

Supplement: S25 Fig — N represents the number of cell cycles extracted from the first cell in a channel of an individual experiment. (PNG) [file pgen.1012110.s027.png]
